# Extracellular peptide production in *Escherichia coli* by inducible downregulation of lipoprotein Lpp via MicL sRNA

**DOI:** 10.1007/s00253-025-13524-z

**Published:** 2025-06-04

**Authors:** Martin Gibisch, Pawel Gorecki, Christopher Tauer, Esther Egger, Matthias Müller, Bernd Albrecht, Rainer Hahn, Gerald Striedner, Monika Cserjan-Puschmann

**Affiliations:** 1 Christian Doppler Laboratory for Production of Next-Level Biopharmaceuticals in 𝐸. 𝐶𝑜𝑙𝑖, Institute of Bioprocess Science and Engineering, BOKU University, Muthgasse 18, 1190 Vienna, Austria; 2 Boehringer-Ingelheim RCV, GmbH & Co KG, Dr.-Boehringer-Gasse 5-11, Vienna, 1120 Austria

**Keywords:** Fed-batch, Outer membrane, Permeability, Knockdown, Secretion, Periplasmic peptides, Leaky strains

## Abstract

**Abstract:**

Despite its many benefits, *Escherichia coli* only poorly secretes recombinant proteins and peptides into the medium. This complicates downstream processing and notably contributes to the production costs of biopharmaceuticals. The permeability of production strains can be increased by deletion of the *lpp* gene, coding for Braun’s lipoprotein Lpp. Consequently, the outer membrane (OM) is destabilized, and periplasmic recombinant proteins/peptides can leak out of the cell into the cultivation medium. However, we observed poor process performance during C-limited fed-batch cultivations in bioreactors when production strains with *lpp* knockout were cultivated. In this study, we developed an inducible system for in-process Lpp downregulation (knockdown) in *E. coli* with the goal to facilitate the release of the periplasmic recombinant fusion peptide CASPON-SST into the cultivation medium. By plasmid-based overexpression of MicL sRNA, we were able to efficiently inhibit Lpp synthesis and increase the OM permeability of our production strains. With this approach, we were able to achieve the secretion of 80–100% of all peptide and increased production capacities. The system was further optimized by utilizing different promoter systems to induce peptide and MicL expression separately in order to coordinate them. We report here for the first time the extracellular production of a recombinant peptide by inducible downregulation of Lpp via MicL sRNA during C-limited fed-batch cultivations. By utilizing a flexible system for Lpp knockdown, potential drawbacks of *lpp* knockout can be counteracted, thus making our approach a valuable tool for the in-process adaptation of OM permeability in production hosts.

**Graphical Abstract:**

**Figure:**
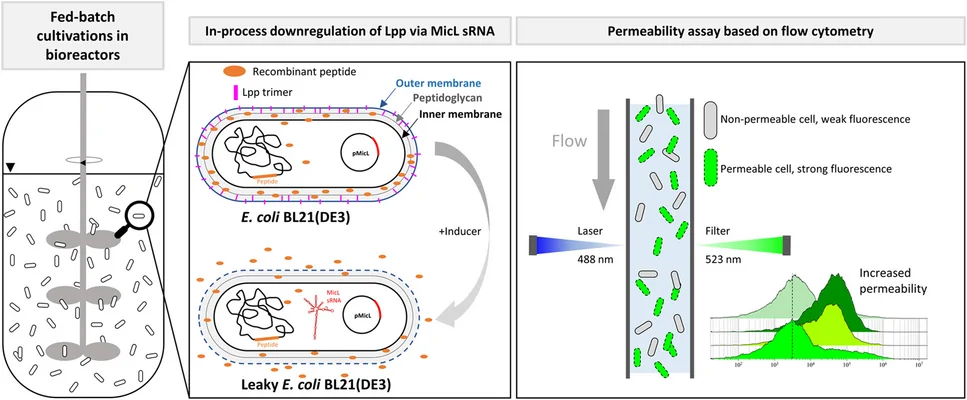

**Key points:**

• *Lpp can be downregulated on mRNA level by MicL sRNA overexpressed during fed-batch.*

• *Recombinant peptides can leak out of the periplasm when Lpp is downregulated.*

• *Leakiness and outer membrane permeability must be distinguished.*

**Supplementary Information:**

The online version contains supplementary material available at 10.1007/s00253-025-13524-z.

## Introduction

The most commonly used bacterium for heterologous gene expression, *Escherichia coli,* produces recombinant peptides and proteins mainly intracellularly. Despite several advantageous characteristics such as rapid growth and high protein yields, its inability to readily secrete recombinant peptides and proteins into the cultivation medium remains a drawback for biopharmaceutical production processes (Kleiner-Grote et al. 2018). This complicates and increases the costs of downstream processing, as the intracellular product needs to be accessed by disrupting the cells and separating the peptide/protein of interest (POI) from host cell-related impurities (Kastenhofer et al. 2021).

Numerous studies have been conducted so far that aimed to increase the outer membrane (OM) permeability of production strains in order to overcome the disadvantage of low secretion efficiencies (Ni and Chen 2009; Kleiner-Grote et al. 2018). Different detergents such as Tween 20, Triton X-100, and polyethyleneimine (PEI), as well as several organic solvents successfully increased the cell permeability for the production of l-carnitine by intracellular enzymes (Cánovas et al. 2005). The peptidoglycan (PG) was also shown to be a promising target for OM permeabilization, as for example the overexpression of D-alanyl-D-alanine carboxypeptidase (DacA) led to increased extracellular GFP production (Yang et al. 2022). Moreover, the supplementation of glycine and glycine in combination with n-dodecane resulted in high extracellular L-asparaginase II production with an increase in extracellular enzymatic productivity, as well as increased vesicle production (Hirayama and Nakao 2020; Flores-Santos et al. 2021). In addition to efforts that aimed to increase the leakiness of the host cell, several studies used protein fusion partners to facilitate the secretion into the medium (Kleiner-Grote et al. 2018). As such, the protein YebF has been reported to be secreted into the medium (Zhang et al. 2006; Arauzo-Aguilera et al. 2023), and was further used to facilitate the secretion of a β-lactamase inhibitor protein (Natarajan et al. 2017). Although systems utilizing YebF are promising, the yields of secreted recombinant protein are oftentimes low, rendering such systems not yet sophisticated enough to be used in industrial bioprocessing (Lokireddy et al. 2025). The prevailing strategy for extracellular protein/peptide production therefore remains adjusting the outer membrane permeability of the host cells.

Strains that leak periplasmic proteins, so-called “leaky” production strains, most often contain mutations in genes that support the structural integrity of the OM. The deletion of the PG-associated lipoprotein (Pal), for instance, resulted in high secretion efficiencies for the production of antimicrobial peptides (Fu et al. 2022). Moreover, the insertion of mature Lpp into the OM was successfully inhibited by supplementing the medium with MAC13243, an inhibitor of the OM lipoprotein carrier protein LolA (Muheim et al. 2017). Despite many potential approaches for increasing the OM permeability exist, most studies evolve around the major OM lipoprotein Lpp. Deletion of the *lpp* gene was previously shown to greatly increase the permeability of the host strain and facilitate the leakage of recombinant proteins into the medium (Koebnik et al. 2000; Ni et al. 2007; Shin and Chen 2008; Chen et al. 2014; Wang et al. 2021), and was even patented (Dassler et al. 2008; Dassler and Kujau 2021).

With up to one million copies in one *E. coli* cell, Lpp is the most abundant protein in numbers. This high abundance was shown to be a result of an AT-rich promoter of the *lpp* gene (Nakamura and Inouye 1979) and an extraordinarily long half-life (~ 12 min) of the Lpp mRNA (Hirashima and Inouye 1973). It is currently the only known lipoprotein that is covalently attached to the PG layer and exists in a 2:1 ratio of bound and free form (Hirashima et al. 1974; Cowles et al. 2011). In its mature form, Lpp is 5.8 kDa in size and usually present in the OM as homotrimers (Braun and Hantke 2019). If Lpp is absent or compromised in its functionality, the structural integrity of the OM appears to be decreased (Mathelié-Guinlet et al. 2020), resulting in an increased OM permeability. Subsequently, this can lead to leakage of periplasmic proteins into the cultivation medium, as well as the increased formation of outer membrane vesicles (OMVs), and blebbing (Asmar and Collet 2018). The Lpp content in the cell is tightly regulated by a σ^E^-mediated stress response. If β-barrel formation or lipopolysaccharide synthesis in the periplasmic space are disturbed, a multi-step pathway is initiated that results in the expression of a small regulatory RNA (sRNA) called MicL, the mRNA-interfering **c**omplementary regulator of Lpp (Klein et al. 2014). In *E. coli*, MicL targets only Lpp mRNA and is the sole regulator of Lpp on translational level. Upon complementarily binding four specific ribonucleotides within the Lpp mRNA sequence, ribosomal translation initiation is inhibited, and the mRNA is degraded. In vivo, the 308 nt MicL transcript is processed to the shorter version MicL-S, consisting of only 80 nt (Guo et al. 2014). The use of MicL for downregulation of Lpp for extracellular protein production was recently published as a preprint by Ahan et al., with the goal of cargo-delivery through membrane-altered *E. coli* Nissle 1917 cells introduced to the human gut (Ahan et al. 2023).

Peptides are amino acid polymers that consist of less than 100 amino acids and are considered “next-level biopharmaceuticals”, as they are becoming increasingly important in the biopharmaceutical landscape due to their versatile applications. Moreover, peptides harbor certain advantages over larger protein-derived therapeutics, making them an attractive alternative to conventional large-size biopharmaceuticals (reviewed by (Lien and Lowman 2003; J. Boohaker et al. 2012; Marqus et al. 2017; Lau and Dunn 2018)). Unlike most proteins, peptides often lack pronounced structural features or tertiary structures. These characteristics result in a strong susceptibility to degradation by host proteases, which is unfavorable especially for recombinant expression in non-native production hosts (Wegmüller and Schmid 2014). To counteract this drawback, peptides are commonly expressed in combination with different fusion partners (Costa et al. 2014). In a previous study, we successfully utilized the novel 4.1 kDa CASPON^®^ tag (Köppl et al. 2022; Lingg et al. 2022) and greatly improved peptide expression during bioreactor cultivations at µL- and benchtop-scale (Gibisch et al. 2024). The CASPON^®^ tag consists of a highly charged, bacteriophage-derived solubility-enhancing sequence (T7AC), combined with a poly-histidine tag for affinity purification, a linker sequence (GSG), as well as a recognition site (VDVAD) for the cleavage by a circularly permutated caspase-2 enzyme (CASPON^®^ enzyme). This system allows the ultrafast and scar-free tag removal before all 20 canonical amino acids. For further details, the reader is referred to (Cserjan-Puschmann et al. 2020; Lingg et al. 2021, 2022; Köppl et al. 2022, 2024; Liu et al. 2024; Müller et al. 2024; Elsner et al. 2025). Among other peptides, somatostatin 1–28 (SST) fused to the CASPON^®^ tag (CASPON-SST) was expressed at high levels up to 2.6 g L^−1^ (Gibisch et al. 2024). SST is a cyclic 28 amino acid peptide hormone with a size of 3.1 kDa. In humans, SST acts as antagonist to growth hormone somatropin, but also inhibits the release of several other hormones (O’Toole and Sharma 2023). Despite high titers being achieved previously, degradation was still evident. This undesirable influence is thought to be eliminated by promoting the release of periplasmic peptides into the cultivation medium. By utilizing production hosts with a destabilized outer membrane, the recombinantly expressed peptides are thought to leak out, thereby preventing degradation by periplasmic proteases. This, alongside other valuable benefits of a membrane-permeable host are thought to greatly improve recombinant peptide production.

The extracellular production of recombinant POIs in *E. coli* remains a challenging topic and is most commonly approached by *lpp* knockout in production strains. Despite the prevailing consensus that *lpp* knockout does not adversely affect growth behavior, however, our experiments conducted with production strains containing Δ*lpp* background showed poor process performance. In this study, we therefore developed an inducible system for Lpp downregulation (Lpp knockdown) via overexpression of the native sRNA MicL during recombinant peptide expression. The MicL system for inducible in-process downregulation of Lpp was implemented during fed-batch cultivations at different scales. Furthermore, to ensure proper coordination of Lpp knockdown and peptide expression, we developed a system for decoupled MicL and peptide expression by using different promoter systems. With our inducible system for Lpp downregulation, we were able to achieve 80–100% release of recombinant peptide into the cultivation medium.

## Materials and methods

### Strains and plasmids

*E. coli* BL21(DE3) was used as chassis for all prepared production strains. The peptide expression cassette was integrated into the attTn7 site in the genome with the previously described “all-in-one” method (Egger et al. 2020). SST was expressed in combination with the N-terminal CASPON^®^ tag (Köppl et al. 2022; Lingg et al. 2022). For posttranslational translocation into the periplasm, CASPON-SST was expressed in combination with an OmpA-derived signal sequence.

MicL sRNA was either expressed from a multi-copy pET30a-derived plasmid or a single-copy pROCOLI-derived (Stargardt et al. 2020) plasmid. The cassette for MicL expression contained either the T7 promoter, repressed by LacI, or the araBAD promoter under control of AraC. The tZENIT terminator (Mairhofer et al. 2014) was inserted at the end of both the peptide and MicL cassettes to terminate transcription. To prevent multimerization, pET30a-derived plasmids contained a *cer* sequence (Summers et al. 1993).

For decoupled expression of peptide and MicL, the arabinose operon (*araABCD*) was deleted from the BL21(DE3) genome in order to prevent the metabolization of arabinose, resulting in the strain BΔ*ara*. This was implemented as proposed by Sharan et al. (2009). The whole operon (between *polB* and *yabI*) was exchanged with a chloramphenicol resistance (Cm^R^, chloramphenicol acetyl transferase) expression cassette flanked by minimal FRT sites. Subsequently, the Cm^R^ cassette was flipped out, leaving behind FRT scars (Datsenko and Wanner 2000). After successful gene knockout, the expression cassette for peptide expression was inserted into the attTn7 site of the newly generated BΔ*ara* strain with the “all-in-one” method. An overview of all plasmids and strains is given in Tables 1 and 2.

**Table 1:** Overview of plasmids used in this study

Plasmid	Description
pET30a*cer*	pET30a plasmid backbone with *cer* sequence to avoid plasmid aggregation and loss. The plasmid contains the *lacI*^*q*^ promoter for enhanced LacI production and a kanamycin resistance. Multicopy ColE1 ori with plasmid copy number of 20–30
pMicL^MC^(lac)	pET30a*cer* plasmid with MicL sequence under control of the T7 promoter and additional *lacO* sequence for gene repression by LacI. MicL is induced by IPTG addition (DE3 system for orthogonal expression). Multicopy ColE1 ori with plasmid copy number of 20–30
pMicL^MC^(ara)	pET30a*cer* plasmid backbone with MicL sequence under control of the *araBAD* promoter. *lacI* exchanged for *araC* for gene repression. MicL inducible by LAra. Multicopy ColE1 ori with plasmid copy number of 20–30
pMicL^SC^(ara)	pROCOLI (Stargardt et al. 2020) plasmid backbone with MicL under control of the *araBAD* promoter. Contains chloramphenicol resistance marker and *araC* for gene repression. Single-copy ori derived from F1-plasmid

**Table 2:** Overview of strains used in this study

Strain	Description
B<oCASPON-SST>	BL21(DE3) with expression cassette for periplasmic CASPON-SST expression integrated to the host genome at the attTn7 site (Gibisch et al. 2024). o: OmpA signal sequence; CASPON: CASPON^®^ tag; SST: somatostatin 1–28: < > : genomic expression
BΔ*ara*	BL21(DE3) with *araABCD* deletion
BΔ*ara*<oCASPON-SST>	BΔ*ara* with expression cassette for periplasmic CASPON-SST expression integrated to the host genome at the attTn7 site
BΔ*ara*<oCASPON-SST> pMicL^MC^(lac)	Production strain containing the multicopy pMicL^MC^(lac) plasmid for coupled peptide expression and MicL co-expression inducible by IPTG
BΔ*ara*<oCASPON-SST> pMicL^MC^(ara)	Production strain containing the multicopy pMicL^MC^(ara) plasmid for decoupled peptide and MicL expression. CASPON-SST expression is induced by IPTG, and MicL expression is induced by LAra
BΔ*ara*<oCASPON-SST> pMicL^SC^(ara)	Production strain containing the single copy pMicL^SC^(ara) plasmid for decoupled peptide and MicL expression. CASPON-SST expression is induced by IPTG, and MicL expression is induced by LAra

### Cultivations

Preculture, main cultures, and sampling were performed as described previously (Weber et al. 2023; Gibisch et al. 2024). Semisynthetic medium with either glucose or glycerol as C-source was used (~ 3 g L^−1^ biomass) for preculture. If strains contained plasmids, kanamycin or chloramphenicol were supplemented at 50 or 20 µg mL^−1^, respectively. After 6–8 h, the main cultures were inoculated. Specific cultivation parameters (inducer, inducer concentration, feed profiles, …) of MTP and STR cultivations are given in the respective sections.

The BioLector™ system (Beckman Coulter GmbH, Germany) was used for cultivations at µL-scale in 48-well microtiter plates. Cultivations were performed with a glucoamylase enzyme (EnPresso Reagent A, EnPresso GmbH, Germany) and 30 g L^−1^ dextran (EnPump 200, EnPresso) for enzymatic glucose-release, resulting in a linear feed profile at constant volume of 800 µL.

For STR cultivations at benchtop scale (1.5 L working volume), the DASGIP® system was used (Eppendorf GmbH, Germany). Cultivations were carried out as fed-batch cultivations with either glucose or glycerol as C-source.

When cultivations were carried out in microtiter plates (BioLector™ system), samples were exclusively drawn at the end of the cultivation, whereas samples were drawn throughout the whole cultivation when using stirred-tank bioreactors (DASGIP® system). The samples that were drawn were samples for gravimetric DCM estimation, 1 mg DCM for enzymatic cell lysis and Lpp-targeted cell lysis, biomass for peptide extraction and HPLC analysis, and cell-free supernatant for analyzing the extracellular peptide and DNA contents.

### Flow cytometry analysis

The OM permeability was monitored by SYTOX™ green uptake and was analyzed via flow cytometry. Samples drawn from STR cultivations were diluted to an OD_600_ of 28 and incubated with 15 µM SYTOX™ green. Samples were diluted 1:2025 in PBS, and flow cytometry measurement was carried out as described before (Schuller et al. 2021). When cultivations were carried out in MTPs, SYTOX™ green was supplemented at 15 µM to the medium from the beginning.

### Cell disruption

Enzymatic cell lysis with lysozyme was performed as described previously (Gibisch et al. 2024). Samples that were destined for Lpp western blot analysis were prepared using an adapted method for Lpp fractionation (Cowles et al. 2011). In short, 1 mg cell pellets were resuspended in PBS containing 1% SDS and boiled at 100 °C for 20 min. Then, each sample was sonicated on ice three times for 60 s using a sonication rod with a cycle time of 0.5 s and an amplitude of 70%. After sonication, 100 µL of a 2 mg mL^−1^ lysozyme stock was added to each sample and incubated over night at 37 °C. On the next day, the samples were centrifuged at 15,000 × g for 10 min. The supernatant, containing the Lpp, was subsequently frozen to −20 °C until further use.

### Cell lysis estimation

DNA in the cell-free cultivation medium was used for estimation of cell lysis (Newton et al. 2016; Fink et al. 2021; Weber et al. 2023). The Qubit 4 fluorometer and respective DNA analysis kits (Invitrogen™, USA) were used for quantification. Cell lysis was estimated according to the specific amount of averagely 20.3 mg DNA per g of BL21(DE3) (Schimek et al. 2020).

1 $$\text{Lysed biomass in }\frac{\mathrm{g}}{\mathrm{L}}= \frac{{\left[DNA\right]}_{\mathrm{extracellular}}\:in\:\frac{mg}{L}}{20.3 \frac{mg DNA}{g DCM}}$$

2 $$\text{Estimated cell lysis in }\%=\frac{\mathrm{Lysed}\; \mathrm{biomass}}{Intact\; DCM+\mathrm{Lysed}\; \mathrm{biomass}}$$

#### RP-HPLC

Peptides were extracted from biomass samples with 2% HCl in extraction buffer (50 mM Tris, 300 mM NaCl, pH = 8.5) at a concentration of 30 g L^−1^ DCM for 3 h at room temperature. After extraction, the samples were centrifuged at 15,000 × g for 10 min, and the supernatants were transferred to a fresh tube. Subsequently, using a micro pH electrode (Mettler Toledo, USA), the pH was neutralized with 5 M NaOH. The samples were filtered through a 0.22 µm filter before RP-HPLC analysis. For detailed information about the procedure, the reader is referred to Müller et al. (Müller et al. 2024).

### SDS-PAGE and western blot

Novex™ Tricine 10–20% mini gels were used for Tricine SDS-PAGE (Schägger 2006) with Coomassie staining for screening of peptide expression and degradation. For western blots, NuPage™ 4–12% Bis–Tris mini gels were used, and further blotted onto a nitrocellulose membrane using the iBlot system and respective materials. All materials (gels, buffers, blotting material) were purchased from Invitrogen™. The cytoplasmic chaperone GroEL was used as loading control and stained with a rabbit anti-GroEL primary antibody (Sigma-Aldrich, USA). Lpp was stained with a Rabbit Anti-Lpp primary antibody generously provided by the research group of Thomas Silhavy (Princeton University, New Jersey, USA). An anti-Rabbit IgG secondary antibody conjugated to alkaline phosphatase produced in goat (Sigma Aldrich, USA) was used as secondary antibody and was stained with BCIP/NBT staining solution (Roche GmbH, Germany).

### RT-qPCR

RNA samples were drawn by first adding 1 mL cell suspension to 0.5 mL of ice-cold stopping solution (5% phenol in EtOH). The stopped cell suspension was further centrifuged (15,000 × g, 10 min, 4 °C) to harvest approximately 3 mg of biomass, which was then frozen in liquid nitrogen and stored at −80 °C. RNA was extracted using the Quick-RNA Fungal/Bacterial Miniprep Kit (R2014, Zymo Research, USA). Purified RNA was then reverse transcribed using the SuperScript™ IV Reverse Transcriptase (Invitrogen™, USA) kit. qPCR was carried out using the iQ™ SYBR^®^ Green Supermix (Bio-Rad, USA) and the MiniOpticon™ system with the CFX™ Manager software. Primer efficiency was tested for all primers by pooling the samples and performing tenfold dilutions for a standard curve. Primer sequences and standard curves are supplemented in Table S1 and Figure S1, respectively. Fold-changes of mRNA levels were evaluated according to the 2^−ΔΔCq^ method (Livak and Schmittgen 2001) with 16S RNA being used as calibrator.

### Mass spectrometry

Mass spectrometry was performed as described before (Gibisch et al. 2024).

### Transmission electron microscopy (TEM)

Cell samples for TEM analysis were prepared as described previously (Jurjevec et al. 2024). In short, cell suspension samples were drawn from STR cultivations. The OD_600_ was adjusted to 0.15–0.2, and the cells were fixed on a pre-treated copper grid with subsequent negative staining. After sample preparation, the cells were analyzed using a TEM FEI Tecnai G2 200 kV (Thermo Fisher Scientific, Waltham, USA) microscope under high vacuum conditions with 160 kV.

## Results

### Influence of MicL co-expression on peptide expression during stirred-tank bioreactor cultivations

**Fig. 1:**
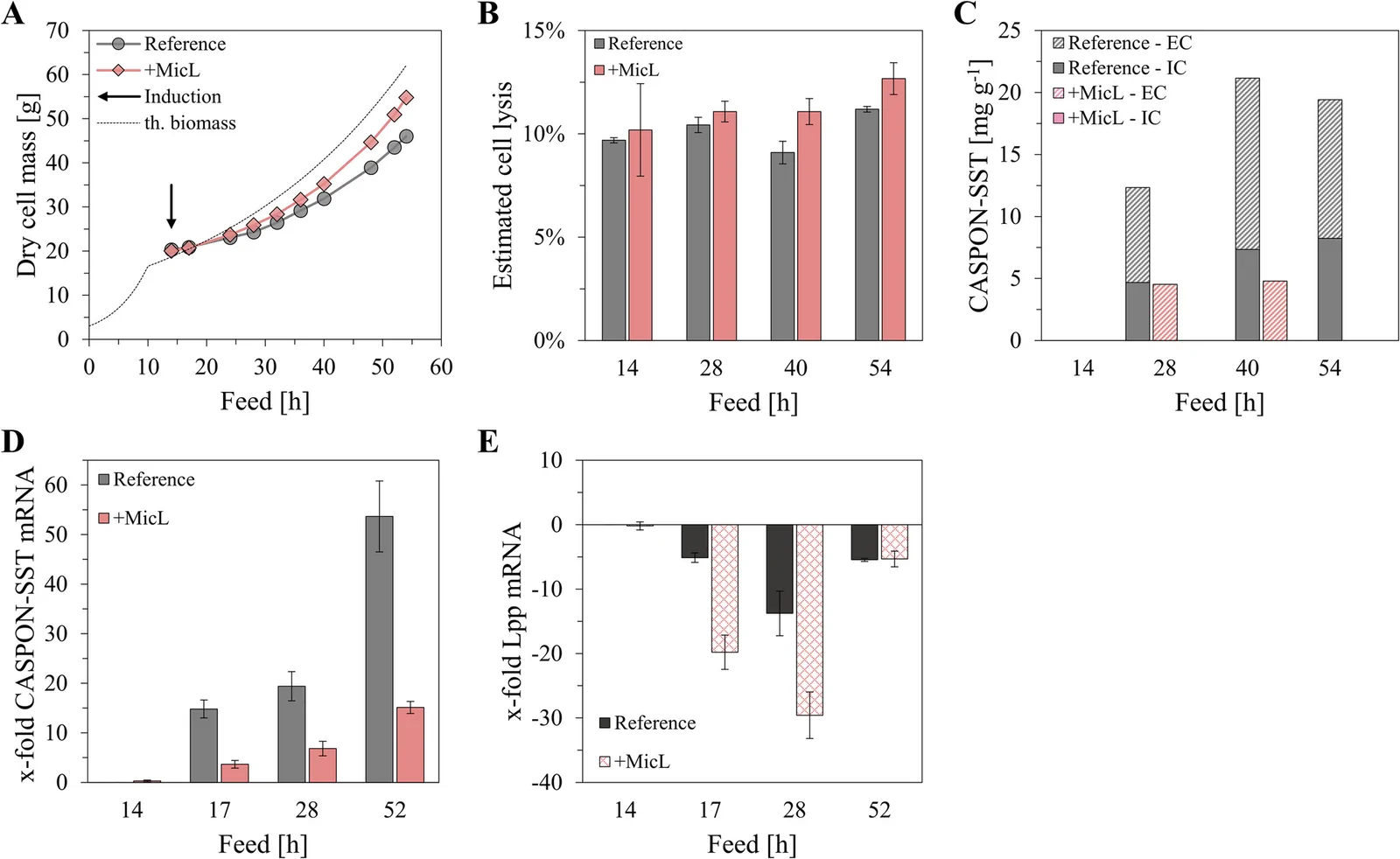
Peptide expression and MicL co-expression (“+ MicL”) from multicopy plasmid in BL21(DE3) during stirred-tank bioreactor cultivations. B<oCASPON-SST> was used as reference, and MicL was co-expressed from the pMicL^MC^(lac) plasmid. **A** Growth curves, **B** estimated cell lysis, **C** specific peptide titer, **D** RT-qPCR analysis of CASPON-SST, and **E** RT-qPCR analysis of Lpp mRNA. The theoretical biomass curve (“th. biomass”, dotted line) was calculated according to the feed profiles with predefined specific growth rates and a Y_X/S_ of 0.4. Cell lysis estimation was evaluated according to measurements of DNA content in the cell-free medium. Peptide content was analyzed via RP-HPLC. All cultures were induced with 2 µmol IPTG per g dry cell mass that was pulsed into the reactors after 14 h (black arrow). EC: extracellular, IC: intracellular. The x-fold mRNA levels were calculated based on the 2^−ΔΔCq^ method, with the reference cultivation before induction being the calibrator for normalization

Initially, we evaluated the impact of *lpp* knockout on the process performance of protein-producing cultures. The *lpp* gene was knocked out in two industry-relevant production strains, BL21(DE3) and HMS174(DE3), where the protein expression cassette was integrated into the host genome. Protein expression with Δ*lpp* background was evaluated during fed-batch processes in stirred-tank bioreactors (STR). Surprisingly, both knockout strains showed poor growth during the feed phase and an overall lower process performance (Figure S2), rendering them unsuitable for production. Note that Fab fragments were expressed as model proteins during these cultivations. Due to the rapidly growing interest in recombinant peptide production in recent years; however, Fab fragments were not considered in future investigations and the priority was changed. In this study, SST (3.1 kDa) served as the model peptide and was expressed as fusion with the N-terminal CASPON^®^ tag (4.1 kDa) in the periplasm of *E. coli* BL21(DE3). The expression cassette was integrated into the host genome, and translocation into the periplasmic space was mediated by the outer membrane porin A (OmpA) signal sequence. This production strain can be referred to as B<oCASPON-SST> (B: BL1(DE3), genomic integration: < >, o: OmpA signal sequence). To promote the release of our model peptide CASPON-SST from the periplasm into the medium while simultaneously maintaining cell growth, we developed an inducible system for Lpp downregulation via MicL. For this, we co-expressed MicL from a pET30a*cer*-derived multicopy (MC) plasmid inducible by IPTG, hereafter referred to as pMicL^MC^(lac). Both CASPON-SST and MicL were under control of the T7 promoter, and additionally repressed by LacI to counteract the basal expression of T7 RNA polymerase (T7 RNAP). We performed STR cultivations as two-step fed-batch cultivations with an initial growth rate of 0.17 h^−1^ that was reduced to 0.03 h^−1^ after 10 h. After 14 h of feed, the cultures were induced with pulsed addition of 2 µmol IPTG per g dry cell mass (DCM). B<oCASPON-SST> was cultivated as reference, and MicL co-expression from the pMicL^MC^(lac) plasmid is referred to as “+MicL” in Fig. 1.

Upon induction, both cultivations deviated from the theoretical growth curve (Fig. 1A). When MicL was co-expressed, cell growth was less impaired compared to the plasmid-free reference. Notably, both cultures lysed to a similar extent (10–12%), shown in Fig. 1B. Soluble intracellular (IC) and extracellular (EC) samples were analyzed via RP-HPLC for peptide quantification. The reference strain produced 19 mg g^−1^ of CASPON-SST after 54 h, with the specific amount being maximal after 40 h (Fig. 1C). Interestingly, roughly 60% of all peptide was extracellular when expressed in the reference strain. The specific peptide titer was strongly reduced when MicL was co-expressed, and over 30% less peptide was produced after 28 and 40 h, respectively. No peptide was detected after 52 h of feed. These findings are reinforced by RT-qPCR analysis, as CASPON-SST mRNA levels were reduced when MicL was co-expressed, compared to the reference cultivation (Fig. 1D). Furthermore, RT-qPCR analysis of Lpp mRNA revealed a ~ 30-fold decrease after 28 h, however, relative Lpp mRNA levels appeared to increase again towards the end of the cultivation (Fig. 1E). After 52 h, the Lpp mRNA level was only fivefold lower compared to the reference before induction. A decrease in Lpp mRNA throughout the cultivation was also observed for the reference strain. After 28 h, the Lpp mRNA was 13-fold lower compared to the reference before induction. Lpp expression was not analyzed on protein level by immunoblotting, as this cultivation strategy was not successful in terms of peptide expression.

### Plasmid-based Lpp downregulation requires higher inducer concentrations for peptide expression

Plasmid-based Lpp downregulation via MicL unexpectedly led to a strong decrease in peptide production. We hypothesized that the negative impact on peptide mRNA levels derived from the presence of the pMicL^MC^(lac) plasmid. The pET30a*cer* plasmid backbone used in this study contains the *lacI*^*q*^ promoter for enhanced LacI production. Amplified LacI levels in the cell caused by high plasmid copy numbers of the pET30a backbone in combination with the *lacI*^*q*^ promoter could result in enhanced repression of the T7 RNAP gene (*lac*UV5 promoter). Furthermore, peptide and MicL expression cassettes contained an additional LacI binding site (*lacO*) downstream of the T7 promoter, contributing to the reduced peptide expression and MicL co-expression. In consequence, the amount of IPTG for induction needs to be increased to compensate for the amplified LacI levels in the cell. We therefore cultivated B<oCASPON-SST> pMicL^MC^(lac) under the same conditions as described before, however, increased the induction levels to 5, 10, and 20 µmol IPTG per g DCM, respectively. Note that B<oCASPON-SST> was not cultivated as reference with higher induction levels. This experiment merely aimed to investigate as to whether higher IPTG levels would restore peptide expression levels and compensate for the increased LacI content in the production strains.

**Fig. 2:**
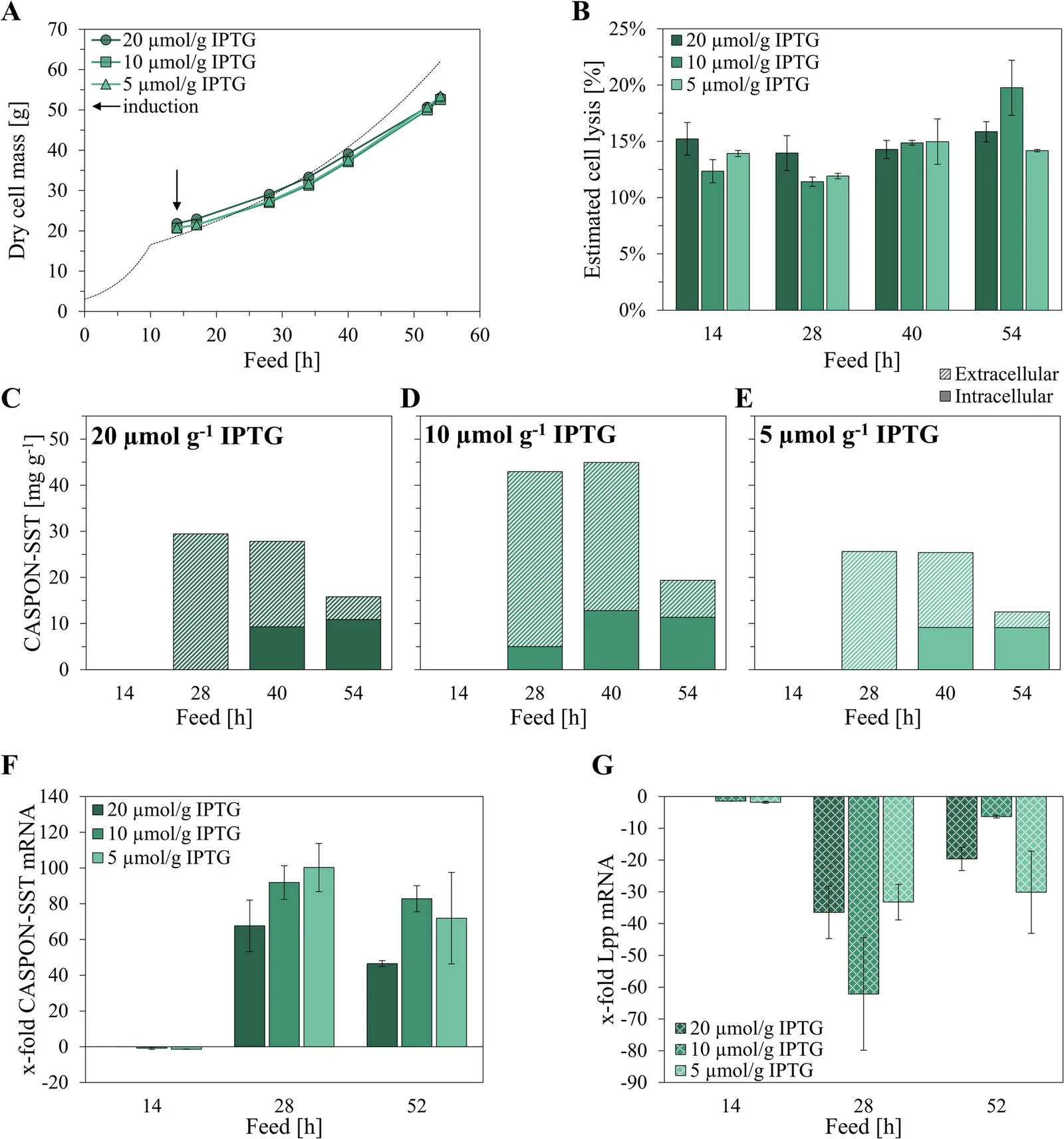
CASPON-SST expression and MicL co-expression during stirred-tank bioreactor cultivations with enhanced induction levels. **A** Growth, **B** estimated cell lysis, **C–E** peptide quantification via RP-HPLC, **F** RT-qPCR analysis of CASPON-SST mRNA levels, **G** RT-qPCR analysis of Lpp mRNA levels. All strains produced the peptide CASPON-SST and co-expressed MicL from the pMicL^MC^(lac) plasmid for Lpp downregulation. Pulsed induction after 14 h is indicated by the black arrow. Cell lysis was estimated according to the DNA content in the cell-free medium. The theoretical biomass curve (dotted line in **A**) was calculated according to a feed profile with predefined specific growth rates and a Y_X/S_ of 0.4. X-fold CASPON-SST mRNA and Lpp mRNA levels were calculated based on the 2^−ΔΔCq^ method, with the uninduced sample of the 20 µmol g^−1^ induction being used as the calibrator for normalization. Error bars represent the standard deviation of triplicates

The growth during the cultivations was comparable to previous cultivations when MicL was co-expressed and did not vary between the different induction levels (Fig. 2A). Cell lysis levels, however, were slightly elevated to 15–20% (Fig. 2B). The specific peptide titer in soluble IC and EC fractions varied between the different inducer concentrations (Fig. 2C–E). When the cultures were induced with 5 and 20 µmol g^−1^ IPTG, 26 and 29 mg g^−1^ CASPON-SST were maximally produced, respectively. With 10 µmol g^−1^ IPTG, a maximum of 45 mg g^−1^ CASPON-SST was produced. Notably, after 28 h of cultivation (14 h induced), up to 100% of all peptides were found to be extracellular. Only a small fraction (12%) of peptide was found in the IC fraction with 10 µmol g^−1^ IPTG. However, the CASPON-SST in the EC fraction decreased over time during all cultivations, and the peptide content in the IC fraction increased. When comparing the CASPON-SST mRNA content between the different induction levels (Fig. 2F), no major differences were observed. Nonetheless, a decrease over time was visible, and the relative mRNA levels were higher after 28 h compared to 54 h. Compared to the previous cultivation induced with 2 µmol g^−1^ IPTG (Fig. 1A), CASPON-SST mRNA levels were increased sixfold. In contrast, Lpp mRNA downregulation was only slightly improved by higher induction levels, as the relative Lpp mRNA levels were similar compared to the cultivation with 2 µmol g^−1^ (Fig. 2G).

**Fig. 3:**
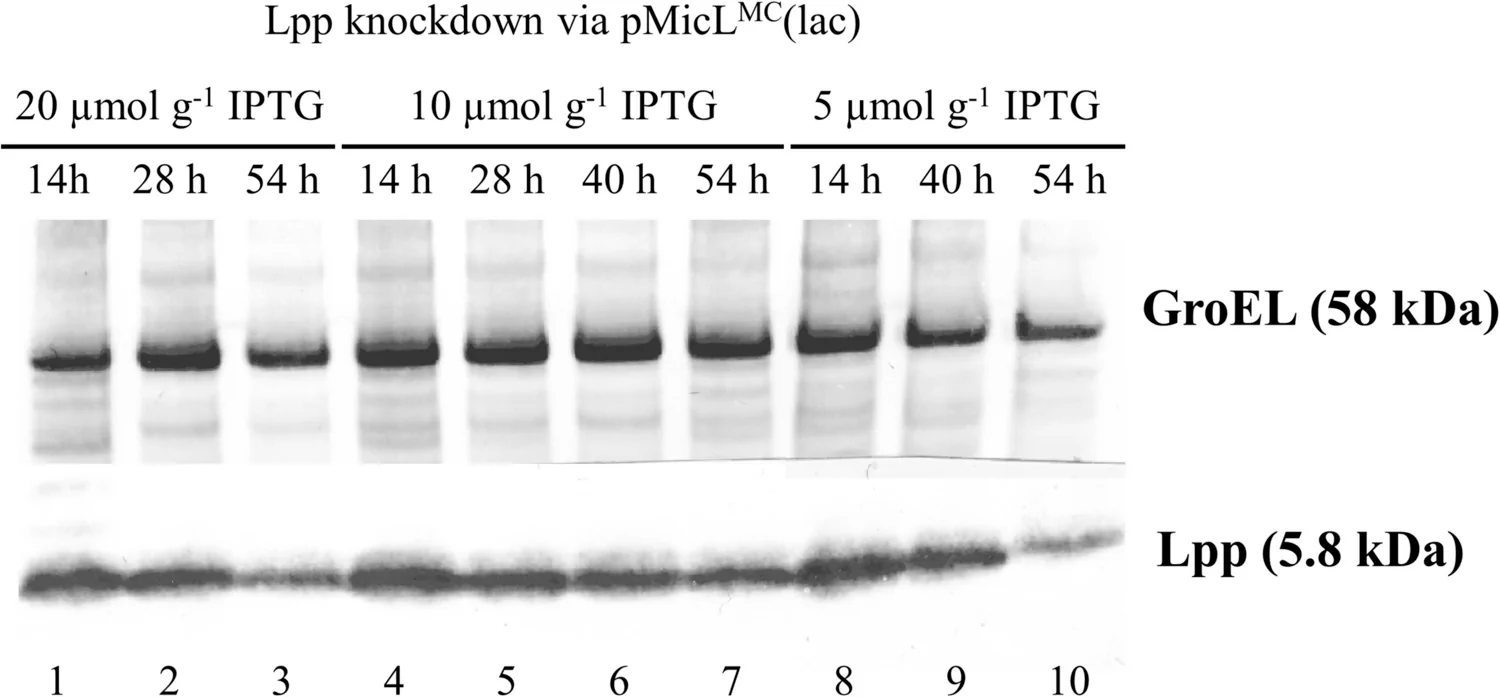
Western blot analysis of intracellular Lpp and GroEL (loading control) during fed-batch cultivations with B<oCASPON-SST> pMicL.^MC^(lac) in stirred-tank bioreactors. Lpp was downregulated via MicL, induced by pulsed addition of IPTG. Different IPTG levels for induction are given as specific concentration in µmol IPTG per g dry cell mass. Lanes 1, 4, and 8 contain uninduced reference samples after 14 h of fed-batch. Note that a slight curvature of the gel resulted in shifted Lpp bands in lanes 8–10

Anti-Lpp western blot revealed Lpp downregulation after 54 h of cultivation (Fig. 3, lanes 3, 7, 10). Note, however, that with 20 and 5 µmol g^−1^ IPTG, the GroEL loading control band appeared weaker compared to the remaining lanes, indicating lower protein loading. Contrary to the results suggested by qPCR analysis, only a slight reduction of the Lpp levels were observed after 28 h. MicL co-expression was induced for 0.4 generations after 28 h, and 1.3 generations after 54 h of feed.

### Decoupled expression of MicL sRNA and peptides increases peptide titers and facilitates peptide release in µ-scale cultivations

In previous experiments, MicL co-expression and peptide expression were induced by the same inducer (IPTG), thereby coupling Lpp downregulation and peptide expression. With this coupled system, it was shown that the Lpp content was only slightly reduced during STR cultivations, and the EC peptide content was only increased initially. We investigated whether a successful Lpp downregulation requires multiple generations of elevated MicL overexpression to achieve sufficient membrane permeabilization and leakage of periplasmic peptides into the cultivation medium. Unfortunately, this poses a major drawback, especially for peptides that exhibit cytotoxic effects on the host cell upon expression, as the production phase must be kept short in order to avoid extensive cell lysis (peptide-dependent). With a coupled MicL/peptide system, however, a long Lpp downregulation phase contradicts a short production phase. To provide a more flexible system for Lpp downregulation, MicL and peptide expression were decoupled. For this, the pMicL^MC^(lac) plasmid used in previous cultivations was adapted as follows: (1) The T7 promoter (P_T7_) upstream of MicL was exchanged for the *ara*BAD promoter (P_BAD_), and (2) the *lacI* coding sequence was exchanged for the *araC* gene under control of a constitutive promoter, resulting in the plasmid pMicL^MC^(ara). Additionally, we aimed to evaluate the gene dosage of MicL needed for Lpp downregulation. For this, we inserted the MicL sequence under control of the P_BAD_ promoter into the single-copy (SC) pROCOLI backbone (Stargardt et al. 2020), resulting in the pMicL^SC^(ara) plasmid. Finally, the metabolization of L-arabinose (LAra) upon induction was avoided by knockout of the *araABCD* genes, creating a Δ*araABCD* background in BL21(DE3), referred to as BΔ*ara*. The cassette for periplasmic CASPON-SST expression was then integrated into the attTn7 site in BΔ*ara* for genomic peptide expression, creating the production strain BΔ*ara*<oCASPON-SST>. This (reference) strain was further transformed with pMicL^MC^(ara) and pMicL^SC^(ara) plasmids, respectively.

The decoupled MicL/peptide systems were initially evaluated in microtiter plates (MTP) using the BioLector™ system for parallel cultivations. Glycerol was chosen as carbon source during batch phase to avoid catabolite repression by glucose and guarantee efficient LAra uptake. LAra was added to the medium at 1 mM before inoculation. After 6.5 h of cultivations, amylase enzyme was added to the cultures for enzymatic glucose release from a dextran polymer (linear feed) at constant volume (800 µL). IPTG was added to the cultures after 16 h at a concentration of 0.5 mM. After 8 further hours, the cultivation was terminated, and samples were drawn. The cultures were induced either with LAra, IPTG, or both LAra and IPTG. Cultivations without any inducer were carried out as reference.

**Fig. 4:**
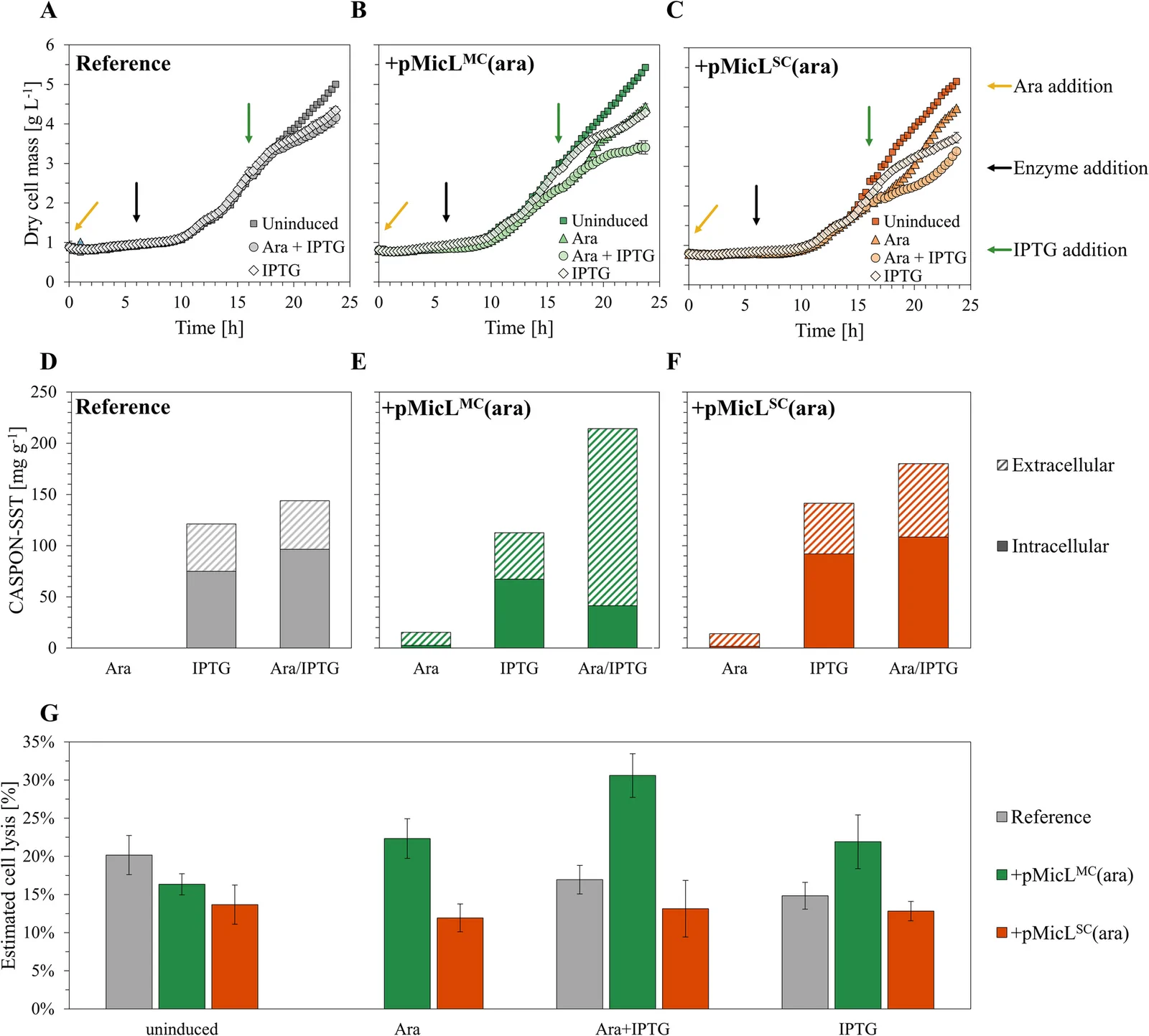
Decoupled peptide and MicL expression during microtiter plate cultivations. **A** Reference cultivation without plasmid. **B** Lpp knockdown via MicL from multi-copy pMicL^MC^(ara) plasmid. **C** Lpp knockdown via MicL from single-copy pMicL^SC^(ara) plasmid. **D–F** Soluble specific intra- and extracellular peptide titer at individual induction. **G** Estimated cell lysis according to DNA content in the cell-free medium. Error bars represent maximum and minimum values of technical duplicates (cell lysis) and biological triplicates (growth curves). MicL expression was induced by LAra addition in batch phase, while IPTG was added to the cultivation after 16 h

Shortly after induction with IPTG, the cultures showed reduced growth rates compared to their respective uninduced reference cultivations (Fig. 4A–C). Differences in growth were even more pronounced when plasmid-carrying strains were induced with LAra, and most notably, with both LAra and IPTG in combination. Shown in Fig. 4D–F, similar IC and EC peptide titers were observed for all strains upon IPTG induction alone (120 mg g^−1^ CASPON-SST, 35–40% extracellular). Induction with LAra and IPTG in combination resulted in slightly elevated IC and EC CASPON-SST contents with both the reference, BΔ*ara*<oCASPON-SST>, and BΔ*ara*<oCASPON-SST> pMicL^SC^(ara). Consequently, the EC peptide content was not increased when MicL was expressed from the single copy plasmid. In contrast, MicL expression from the multicopy pMicL^MC^(ara) plasmid resulted in 48% and 19% higher specific peptide titer compared to the plasmid-free reference and BΔ*ara*<CASPON-SST> pMicL^SC^(ara), respectively. With the pMicL^MC^(ara) plasmid, 81% of all peptide was extracellular. Notably, the estimated cell lysis levels (Fig. 4G) were similar for all cultivations, and only a slightly elevated cell lysis level was observed for BΔ*ara*<oCASPON-SST> pMicL^MC^(ara).

**Fig. 5:**
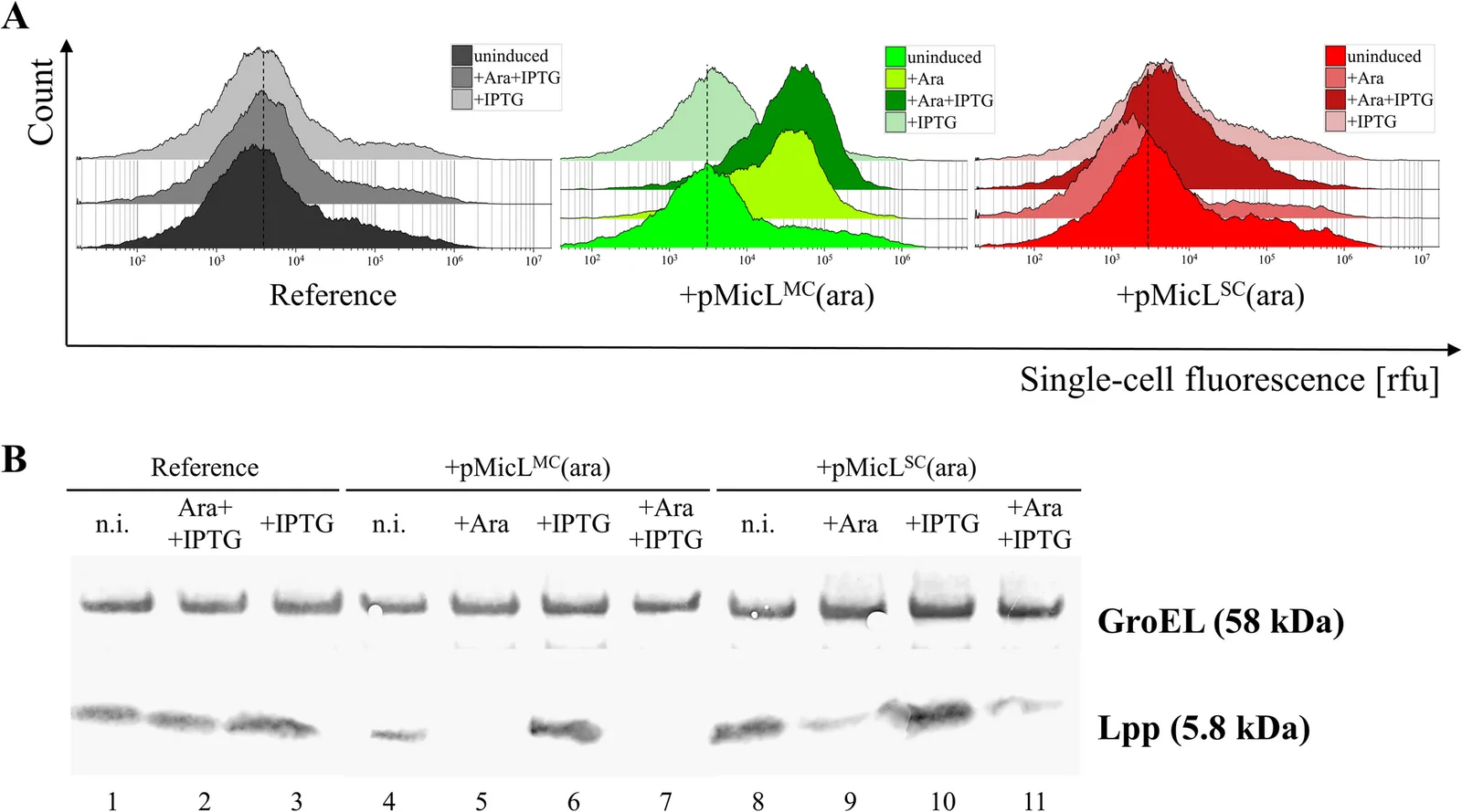
**A** Flow cytometry analysis of strains downregulating Lpp during microtiter plate cultivations. **B** Anti-Lpp western blot analysis of end-point samples after the cultivation. GroEL was used as loading control. Uninduced samples are indicated as “n.i.” (not induced). Induction with LAra and IPTG induction is indicated as “+Ara” and “+IPTG”

To monitor the effects of Lpp downregulation on the OM permeability, the fluorescent DNA dye SYTOX™ green was supplemented to the cultivation medium as a permeability indicator. In theory, an increased OM permeability should result in an increased SYTOX™ green uptake. An increased fluorescence signal therefore should indicate an increased OM permeability. After the cultivations, the cell suspensions were diluted and subsequently analyzed via flow cytometry. Induction with LAra resulted in increased single-cell fluorescence (Fig. 5A). This effect, however, was only pronounced with the pMicL^MC^(ara) plasmid. In contrast, when MicL was overexpressed from the pMicL^SC^(ara) plasmid, only a minor increase in fluorescence was observed that was also visible when induced with IPTG alone. These results are in accordance with anti-Lpp western blot analysis (Fig. 5B), as the Lpp content was strongly reduced with pMicL^MC^(ara). However, Lpp levels were only slightly reduced with pMicL^SC^(ara) when induced with LAra. In the reference strain, the Lpp content appeared constant independent of inducer.

### Evaluation of decoupled MicL/peptide system in stirred-tank bioreactors

The system for decoupled Lpp downregulation via MicL and peptide expression was transferred to stirred-tank bioreactors, and fed-batch processes were performed. BΔ*ara*<oCASPON-SST> was cultivated as reference, and MicL was either expressed from pMicL^MC^(ara) or pMicL^SC^(ara), respectively. Glycerol was used as C-source for both batch and fed-batch phase. MicL expression was induced after 5 h of batch phase by addition of LAra (10 mM). After 12 h, an exponential feed with a specific growth rate, µ, of 0.03 h^−1^ was started and maintained for 52 h (48 g DCM final theoretical DCM). Peptide expression was induced by pulsed addition of 2 µmol IPTG per g DCM (specific to biomass at the end of the cultivation).

**Fig. 6:**
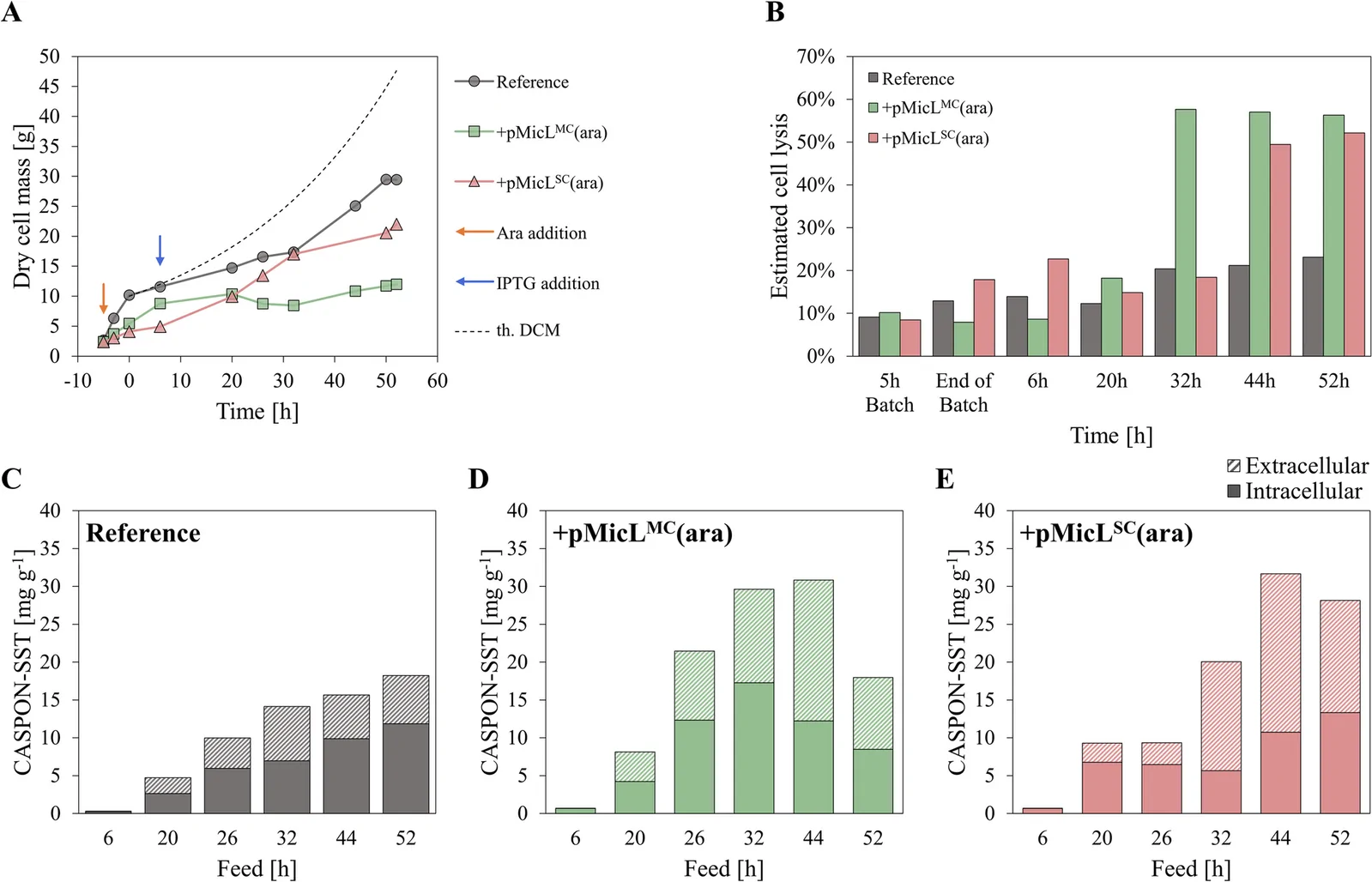
STR cultivations with decoupled MicL/peptide system. MicL was either expressed from multicopy (green) pMicL^MC^(ara) or single-copy (red) pMicL^SC^(ara) plasmid. BΔ*ara*<oCASPON-SST> was cultivated as reference. **A** Growth curves, **B** estimated cell lysis, **C**, **D**, **E** specific peptide titer. Cell lysis was estimated based on the DNA content in the cell-free supernatant. Peptide content was estimated via quantitative SDS-PAGE analysis. The theoretical growth curve (“th. DCM”) was calculated according to a batch biomass of 10 g and a µ of 0.17 and 0.03 h^−1^, respectively. Cultures were induced with LAra after 5 h during batch phase. IPTG was added to the cultivation after 6 h of feed

Induction with LAra showed a strong negative effect on growth whenever the host strains carried a plasmid (Fig. 6A), while the reference was not visibly affected. A strong decline in growth was visible even shortly after induction with LAra; however, cell lysis levels were comparable among all strains up to 20 h of feed. Plasmid-carrying strains only lysed to a stronger extent after 32 and 44 h, respectively (Fig. 6B). The intra- and extracellular peptide contents were analyzed via RP-HPLC after treatment with HCl. Unexpectedly, results obtained via RP-HPLC were inconclusive, as almost no extracellular CASPON-SST could be identified. This contrasted with SDS-PAGE analysis, where clear bands ascribed to CASPON-SST were visible (Supplementary information, Figure S3). Therefore, we chose to quantify the IC and EC CASPON-SST content via SDS-PAGE using purified peptide standards. A total specific CASPON-SST titer of 18.2 mg g^−1^ (500 mg L^−1^), was reached with the reference after 52 h of feed, with a steady increase in IC/EC peptide content throughout the cultivation. BΔ*ara*<oCASPON-SST> pMicL^MC^(ara) produced a total of 30.8 mg g^−1^ (335 mg L^−1^) after 44 h; however, this decreased to 18 mg g^−1^ (216 mg L^−1^) after 52 h. BΔ*ara*<oCASPON-SST> pMicL^SC^(ara) produced 31.7 mg g^−1^ (433 mg L^−1^) in total after 44 h; however, again, this declined slightly to 28.2 mg g^−1^ (428 mg L^−1^). On average, the total specific peptide titers were 1.9- and 1.7-fold higher when MicL was expressed, respectively. Moreover, the relative EC peptide content was nearly constant at ~ 40% over time in the reference cultivation, while 50–70% of all peptide was extracellular when Lpp was downregulated. With the single-copy plasmid, 70% of all peptide was found extracellular after 32 h despite cell lysis levels being low.

**Fig. 7:**
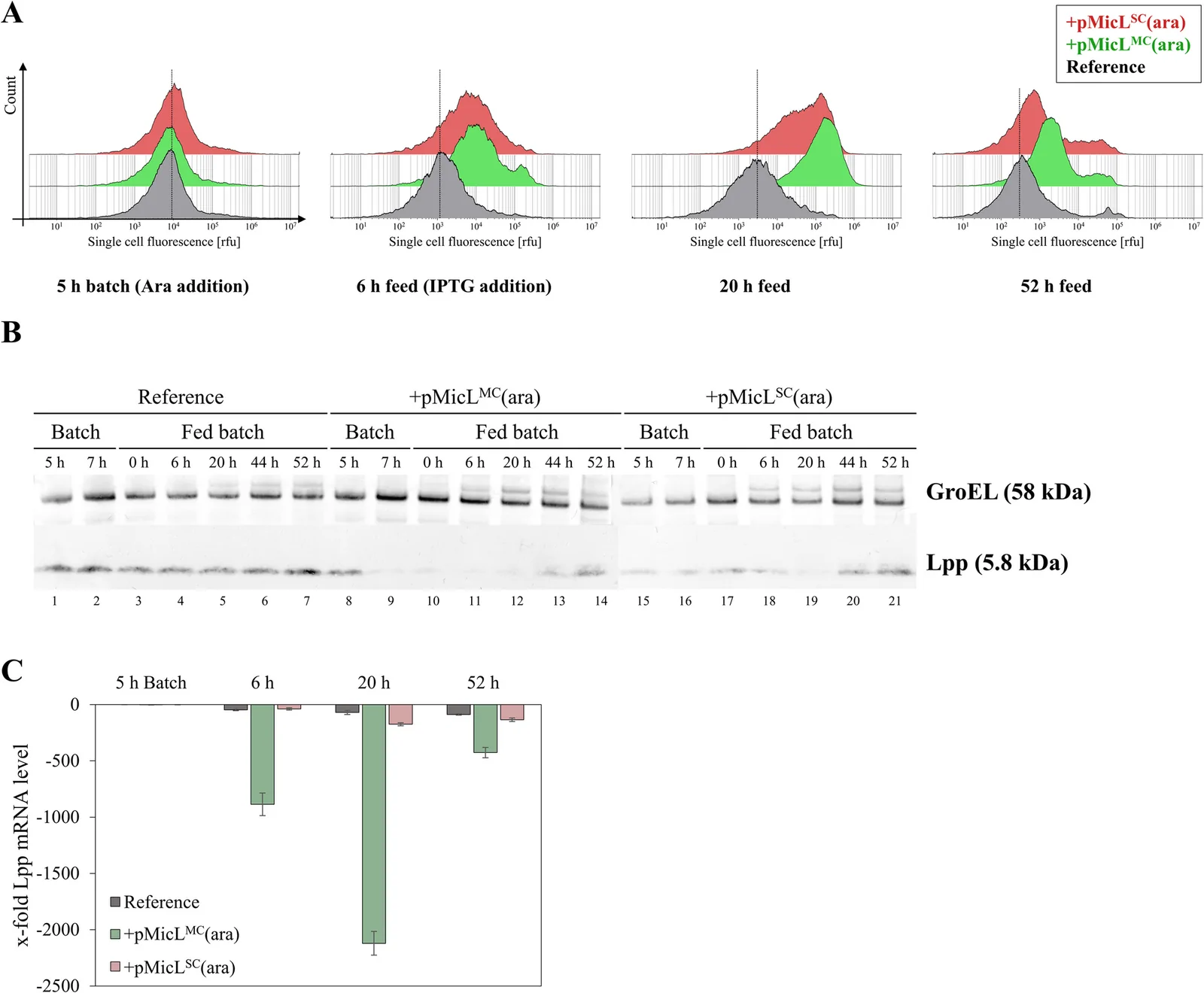
**A** Flow cytometry, **B** western blot, **C** and RT-qPCR analysis of intracellular Lpp content in cells expressing MicL during fed-batch stirred-tank bioreactor cultivations. MicL expression was induced by the addition of LAra after 5 h in batch phase. Peptide expression was induced after 6 h in fed-batch by addition of IPTG. For flow cytometry analysis of single-cell fluorescence, cell suspension samples were incubated with 15 µM SYTOX™ green for 1 min before dilution in PBS and subsequent flow cytometry. Dotted lines in **A** indicate the single-cell fluorescence values of the reference cultivation at the maximum population density. GroEL in **B** was used as loading control in Anti-Lpp western blots. x-fold Lpp mRNA levels in **C** were estimated based on the 2^−ΔΔCq^ method, where the reference cultivation before induction (5 h batch) was used as calibrator. Error bars represent the standard deviation of triplicates

The OM permeability was analyzed by incubating the cell suspension at different timepoints with SYTOX™ green and subsequent flow cytometry analysis (Fig. 7A). After 6 h of feed (11 h after MicL induction with LAra), a tenfold increase in single-cell fluorescence was visible. This increase in SYTOX™ green uptake was even stronger after 20 h of feed, where BΔ*ara*<oCASPON-SST> pMicL^MC^(ara) and BΔ*ara*<oCASPON-SST> pMicL^SC^(ara) both showed ~ 100-fold higher single-cell fluorescence signals compared to the reference. Over time, the OM permeability decreased, thereby showing lower single-cell fluorescence values for the plasmid-carrying strains.

The results obtained by flow cytometry analysis were complemented by anti-Lpp western blot and RT-qPCR of Lpp mRNA levels. After induction with LAra, the Lpp content was strongly reduced (Fig. 7B). Interestingly, a strong reduction of Lpp was observed after 2 h of induction with LAra, suggesting that Lpp was effectively downregulated even after only a short time of MicL overexpression. Especially with the multi-copy pMicL^MC^(ara), Lpp mRNA was downregulated over 2000-fold after 20 h of feed (Fig. 7C). A reduction of Lpp on protein and mRNA level was also visible with the single-copy pMicL^SC^(ara) plasmid. With the single-copy plasmid, however, Lpp was still visible on the western blot throughout the cultivation, and RT-qPCR revealed that the downregulation on mRNA level was less effective compared to the multi-copy plasmid.

Although the proof of principle for Lpp downregulation was confirmed when cultivations were performed in the STR system, an increased extracellular peptide content was not achieved. The specific peptide titer was increased; however, the volumetric titers were lower compared to the reference due to the lower biomass of MicL-overexpressing strains. We therefore attempted to counteract the negative influence of Lpp downregulation on cell growth by adapting the process (feed profile, inducer concentrations, order of LAra/IPTG addition, induction timepoints). Unfortunately, the outcomes were similar to what was shown in the previous cultivations, and the attempts to optimize the process were not successful. The different cultivation strategies are shown in Figure S4.

### Leaky strains exhibit elevated levels of extracellular outer membrane proteins

**Fig. 8:**
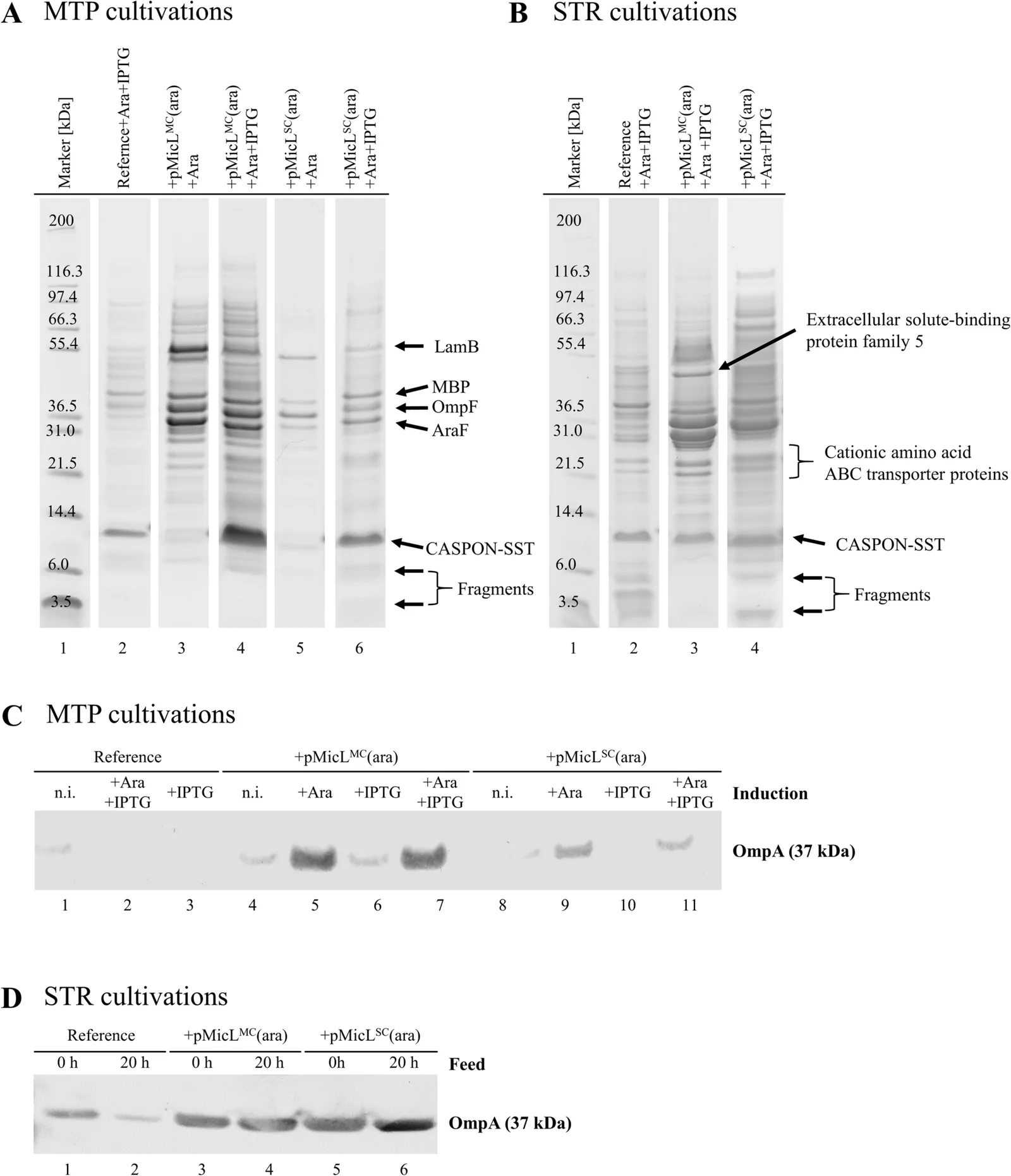
Detection of outer membrane proteins in extracellular samples derived from MTP and STR cultivations where Lpp was downregulated via MicL. **A**, **B** SDS-PAGE analysis of extracellular samples from MTP and STR cultivations; **C**, **D** anti-Lpp western blot analysis of extracellular samples from MTP and STR cultivations. The strain BΔ*ara*<oCASPON-SST> served as reference, and MicL was either expressed from pMicL^MC^(ara) or pMicL^SC^(ara). Arrows in panels **A** and **B** indicate proteins analyzed via mass spectrometry. Samples for western blot analysis were diluted to normalize protein loading

When Lpp was downregulated to a high extent, RP-HPLC analysis of extracellular samples was oftentimes inconclusive. Almost no peptide was detected in these samples via RP-HPLC, whereas in SDS-PAGE clear CASPON-SST bands were visible (exemplarily shown in Figure S3). Hardware-related errors were ruled out, as RP-HPLC analysis of samples spiked with purified CASPON-SST gave clear peaks (data not shown). Alterations in the outer membrane composition of *E. coli* (and other microbes) are known to cause the formation of outer membrane vesicles (OMVs) and/or blebbing. Our previous results showed that the Lpp content was greatly reduced upon MicL expression with the decoupled MicL/peptide system. This suggests that OMVs could have formed during the cultivation, which could lead to difficulties in peptide detection with chromatography-based methods. Note that due to insufficient Lpp downregulation with the coupled MicL/peptide system (both inducible by IPTG), only the decoupled system (induced with LAra and IPTG) is covered here. The cell-free supernatants (after centrifugation) of BΔ*ara*<oCASPON-SST>, BΔ*ara*<oCASPON-SST> pMicL^MC^(ara) and BΔ*ara*<oCASPON-SST> pMicL^SC^(ara) during MTP and STR cultivations were analyzed via SDS-PAGE and western blot. When BΔ*ara*<oCASPON-SST> pMicL^MC^(ara) and BΔ*ara*<oCASPON-SST> pMicL^SC^(ara) were cultivated and induced with LAra or LAra and IPTG in combination (Fig. 8A), three distinct bands at ~ 37 kDa and two distinct bands at ~ 55 kDa were visible. While these bands were also visible when the reference strain was cultivated, they were considerably more distinct when Lpp was downregulated. Notably, cell lysis levels were low for all cultivations, hence, the bands visible in the extracellular space are thought to be a result of a destabilized membrane due to Lpp downregulation. During STR cultivations, the same distinct bands were visible whenever Lpp was downregulated (Fig. 8B). Two additional bands at ~ 22 kDa were clearly visible, however, were also present in the cell-free supernatants of the reference strain. The bands were cut out of the respective lanes/gels and analyzed via mass spectrometry (LC–ESI–MS/MS). Indicated as black arrows in Fig. 8A, the proteins were identified as maltoporin (LamB, Figure S5), extracellular solute-binding protein family 5 (Figure S6), maltose binding protein (MBP, Figure S7), outer membrane porin F (OmpF, Figure S8), LAra-binding periplasmic protein (AraF, Figure S9), and two cationic amino acid ABC transporter proteins (similar sequence for both bands, shown in Figure S10). The overabundance of outer membrane proteins clearly indicates the formation of OMVs. Therefore, we performed western blots against OmpA, a commonly used protein marker for OMVs, in extracellular cultivation samples. When Lpp was downregulated during MTP cultivations, the OmpA content in the cell-free supernatant was strongly increased compared to the reference. However, more extracellular OmpA was found with pMicL^MC^(ara) compared to pMicL^SC^(ara) (Fig. 8C). During STR cultivations, OmpA was also found in cell-free supernatant samples of the reference. Nonetheless, the extracellular OmpA contents in cell-free supernatant samples of BΔ*ara*<oCASPON-SST> pMicL^MC^(ara) and BΔ*ara*<oCASPON-SST> pMicL^SC^(ara) were strongly increased compared to the reference (Fig. 8D), thus complementing the results obtained from MTP cultivations.

Interestingly, proteolysis was seemingly decreased when Lpp was downregulated. Distinct CASPON-SST degradation fragments were visible below the intact peptide, when expressed in BΔ*ara*<oCASPON-SST> and BΔ*ara*<oCASPON-SST> pMicL^SC^(ara) (Fig. 8A, B, bands at 3–6 kDa indicated as fragments). This observation could be made independent of scale. However, when Lpp was downregulated via pMicL^MC^(ara), the degradation fragments appeared to be almost absent. This effect was more pronounced during STR cultivations, as during MTP cultivations the CASPON-SST degradation fragments were far less distinct even in the reference cultivation without Lpp downregulation.

### Visualization of outer membrane vesicles via transmission electron microscopy

The elevated amount of extracellular OM proteins at low cell lysis levels indicated the formation of OMVs. We therefore analyzed the cell suspensions of STR cultivations via transmission electron microscopy (TEM). The analyzed samples derived from cultivations with a linear feed profile (Figure S4D) and were drawn at the end of the cultivation. MicL was expressed from the pMicL^MC^(ara) plasmid during the cultivations, and BΔ*ara*<oCASPON-SST> was cultivated as reference.

**Fig. 9:**
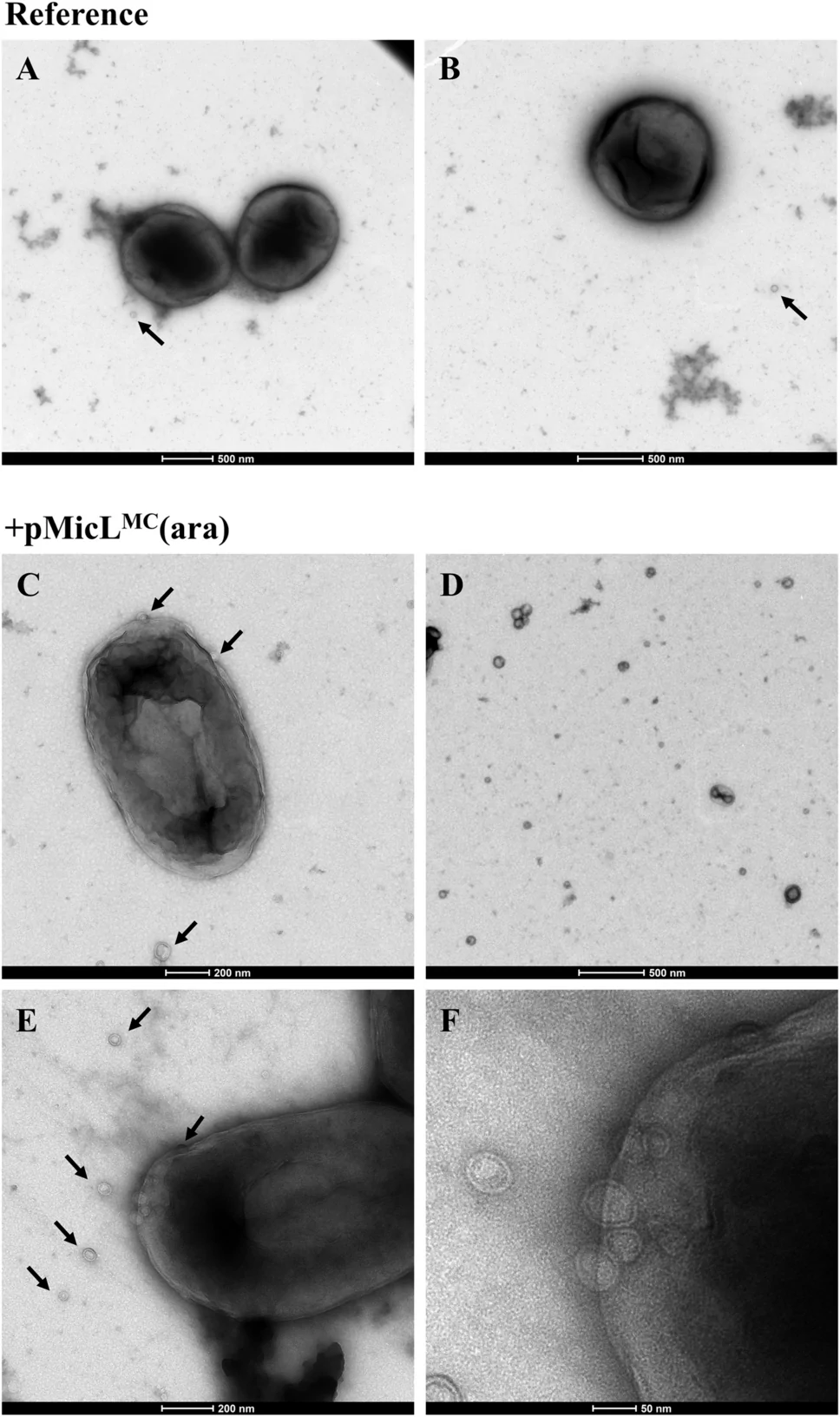
Transmission electron microscopy of membrane-altered cells during STR cultivations. **A**, **B** BΔ*ara*<oCASPON-SST> pMicL^MC^(ara) cells as control without Lpp downregulation during linear feed, **C–F** BΔ*ara*<oCASPON-SST> pMicL^MC^(ara) cells during linear feed. Cells were induced with LAra and IPTG for induction of MicL expression and recombinant peptide production, respectively. Black arrows indicate outer membrane vesicles

When cultivating the reference strain, the cells were spherical and exhibited a smooth surface (Fig. 9A, B). OMVs were detected, albeit only occasionally. When Lpp was downregulated, the cells were rod-shaped, however, exhibited a wavy and inconsistent surface (Fig. 9C). OMVs were detected near the cells but were also randomly scattered across the sample grid (Fig. 9D). Moreover, OMVs were predominantly detected at the cell poles and rarely visible at the sides (Fig. 9E, F).

TEM imaging confirmed the formation of OMVs when Lpp was downregulated. Western blot analysis targeting Lpp and flow cytometry analysis complements these observations, as the Lpp content decreased over time when induced with LAra, while the single-cell fluorescence signals (SYTOX™ green uptake) steadily increased towards the end of the cultivation (Figure S11). Interestingly, OMVs were visible although the remaining Lpp content throughout the cultivation was higher compared to previous cultivations.

## Discussion

The major OM lipoprotein Lpp plays an important role in the stability of the cell envelope and has been subject of numerous studies that focus on extracellular protein production (Kleiner-Grote et al. 2018). The absence of Lpp was shown to have a destabilizing effect on the OM, thus making the production strain leaky (Ni et al. 2007; Ni and Chen 2009; Chen et al. 2014). Consequently, periplasmic proteins can be released across the OM into the cultivation medium. In the context of bioprocesses for the production of biopharmaceuticals, leaky production hosts harbor an economic advantage over conventional bioprocesses, as they reduce the costs for extraction and purification (Kastenhofer et al. 2021). In preliminary experiments, we found that the process performance (cell growth and protein production) of production strains with Δ*lpp* background was subpar compared to reference cultivations. This negative impact on the process performance was evident for production strains derived from both *E. coli* B and *E. coli* K-12 (Figure S2).

The main objective of this study was to develop an inducible system for Lpp downregulation as an alternative to a knockout strategy. With this knockdown approach, we intended to destabilize the OM via plasmid-based overexpression of the small regulatory sRNA MicL, a native sRNA that solely inhibits the translation of Lpp mRNA (Guo et al. 2014). We expressed the peptide SST in combination with the N-terminal CASPON^®^ (Lingg et al. 2021, 2022; Köppl et al. 2022) tag as model peptide to evaluate the peptide release into the extracellular cultivation medium. The peptide expression cassette was integrated into the genome of the *E. coli* host BL21(DE3), while MicL was co-expressed from the pET30a*cer*-derived multicopy plasmid pMicL^MC^(lac). Both peptide and MicL were under control of the T7 promoter and inducible by IPTG, hence coupling the system. During STR cultivations with pulsed addition of 2 µmol IPTG per g DCM, we investigated the influence of MicL co-expression on cell growth and CASPON-SST production. Upon MicL co-expression, the peptide titers were drastically reduced compared to the reference cultivation (Fig. 1), and only a small amount was found in the extracellular compartment. The plasmid for MicL co-expression contained the *lacI* gene for repression under control of the *lacI*^*q*^ promoter. In consequence, we assumed that the LacI content in the plasmid-carrying strain was too high to enable proper peptide expression, especially since only one copy of the recombinant peptide gene was present in the host genome. We therefore repeated the cultivations, and both peptide and MicL were induced with increased IPTG concentrations (5, 10, and 20 µmol IPTG per g DCM). With the increased IPTG concentrations, peptide production was restored and even increased compared to the reference cultivation induced with 2 µmol g^−1^ IPTG. Moreover, after 28 h of feed (14 h induced, 0.4 generations), up to 100% of all peptide was found extracellular (Fig. 2). The orthogonal DE3 system contains T7 RNAP under control of the *lacUV5* promoter, thereby being repressed by the LacI repressor protein (Gopal and Kumar 2013). *E. coli* naturally expresses LacI; however, only two copies of the *lacI* gene are present in the host genome, and the resulting LacI content is low (Semsey et al. 2013). A pET30a-derived plasmid was used for MicL co-expression, thus increasing the gene dosage of *lacI* more than tenfold due to constitutive LacI expression by the strong constitutive *lacI*^*q*^* promoter*. In combination with the increased amount of LacI in the host strain and the fact that an additional *lacO* sequence (LacI binding site) were present in the peptide expression cassette, the expression levels were diminished. Consequently, the specific and volumetric peptide titers were decreased, which was confirmed by RT-qPCR analysis of the CASPON-SST mRNA levels (Fig. 1D). When induced with higher IPTG concentrations, the CASPON-SST mRNA levels were restored, and the specific peptide titer could be compensated (Fig. 2F). Notably, however, peptide expression levels were not constant and decreased over time. We believe this to be a result of pulsed IPTG addition into the reactors for peptide/MicL induction, which resulted in a decrease of both the specific and volumetric IPTG concentrations over time. The IPTG was being continuously bound by LacI until the levels of free-form LacI exceeded the levels of bound-form LacI and repressed peptide expression. This had an inverse effect on the Lpp mRNA levels analyzed via RT-qPCR. After 28 h of feed, Lpp mRNA levels were 30–60 times lower compared to the uninduced reference when MicL was co-expressed; however, the Lpp mRNA levels increased over time due to continuous repression of the *micL* gene. The MicL expression levels not being stable throughout the cultivation could be a result of increased LacI levels in the cell, however, could also derive from plasmid loss. Plasmid loss was not analyzed and deemed unlikely due to the presence of a *cer* sequence on the plasmid for enhanced plasmid stability (Summers and Sherratt 1988), previously also proven to increase the plasmid stability during C-limited cultivations (Allen et al. 2022).

Interestingly, the Lpp mRNA levels were also 14-fold lower in the reference strain (2 µmol IPTG per g DCM) after 28 h of feed (Fig. 1E). This indicates that the process conditions and/or peptide expression can impact Lpp expression, thus might influence the integrity of the cell envelope even without active cell manipulation. Lpp is under tight control of the σ^E^-mediated stress response (Guo et al. 2014; Grabowicz and Silhavy 2017), which can influence the cell globally. We therefore believe that compromised Lpp mRNA levels derived from recombinant expression-mediated stress responses. Previously, we analyzed cell integrity via atomic force microscopy and found that the cells were softer and more fluid-like when CASPON-SST was expressed (Weber et al. 2023), thereby proposing a possible explanation to our findings. This agrees with previously published data, where the absence of Lpp was shown to result in softer, less rigid cells (Mathelié-Guinlet et al. 2020). Moreover, it was shown previously that low growth rates can lead to increased polyadenylation of Lpp mRNA, thereby being degraded more efficiently (Jasiecki and Wegrzyn 2003). The applied feed rate in our STR cultivations was set to a relatively low specific growth rate (µ = 0.03 h^−1^) and therefore could have impacted Lpp mRNA levels. Even when MicL was not overexpressed, high relative extracellular peptide contents were observed here and in previous studies (Weber et al. 2023; Gibisch et al. 2024). In view of the data presented here, our previous findings could have resulted from a diminished OM integrity. It must be noted, however, that CASPON-SST also showed strong signs of proteolytic degradation. A high extracellular/intracellular peptide ratio therefore also could have been a result of low intracellular peptide content due to proteolytic degradation of the peptide. This can be reinforced by the fact that peptides/proteins are less susceptible to degradation in the extracellular space (Maurizi 1992; Gottesman 1996), under the premise that cell lysis levels are low.

Even at high inducer concentrations, Lpp was not downregulated to a high extent on protein level, despite Lpp mRNA levels were reduced up to 60-fold (Fig. 3). Lpp naturally exists as a free and bound form. The free form was shown to span the OM and being exposed to the extracellular space, while the bound form is exposed to the periplasmic space and covalently bound to the PG layer. Being exposed to the periplasm, the bound form can be degraded by proteases, while the free form cannot. Moreover, the free and bound form (1:2 ratio) were shown to be in a dynamic equilibrium (Inouye et al. 1972; Cowles et al. 2011), meaning that decreased Lpp mRNA levels would not necessarily result in the complete absence of Lpp on protein level. However, with a reduced synthesis rate due to lowered amounts of Lpp mRNA, periplasm-exposed Lpp would be degraded, and the covalent linkage to the PG would be reduced. This in turn would result in a slightly destabilized membrane even though the majority of Lpp is still present in the cell. As indicated by our results, peptide leakage into the medium could thereby be achieved even though the majority of Lpp was still present after a short time. As discussed before, the increased relative EC peptide content might also derive from strong proteolysis of IC peptide. Under the assumption that Lpp translation was efficiently inhibited by MicL, however, the reduced Lpp synthesis rates should have resulted in a “thinning out” effect of the remaining Lpp in the cell upon duplication. This was not the case when MicL expression was coupled to peptide expression independent of inducer concentrations (Fig. 3). Given the strong gene repression by LacI, we therefore believe that the Lpp mRNA transcription rates still exceeded those of MicL, especially when considering the strong Lpp promoter (Nakamura and Inouye 1979), high intracellular LacI contents, and the decrease of specific IPTG concentrations over time.

The coupled MicL/peptide system was evidently only successful for a short time (0.4 induced generations), as Lpp was not consistently downregulated on mRNA and protein level (Figs. 1, 2). Moreover, OmpA (~ 200,000 copies), as well as the PG-associated lipoprotein Pal (~ 60,000 copies) were shown to non-covalently bind the PG layer, thereby contributing to the structural integrity of the cell (Chen et al. 2014). We therefore hypothesized that a prolonged Lpp knockdown phase would be required to sufficiently decrease the Lpp content and reliably promote the leakiness of the production strain. Unfortunately, the production of certain peptides can lead to excessive cell lysis (Gibisch et al. 2024), which would nullify an increased OM permeability. We therefore wanted to increase the flexibility of the system and aimed for a more efficient coordination of peptide and MicL expression to optimize the effects of a leaky OM on extracellular peptide production. For this, MicL and peptide expression were decoupled. The ara operon was used for MicL expression under control of the *ara*BAD promoter (induction by LAra), and peptide expression was induced by IPTG (orthogonal DE3 system). For a proof of concept, the decoupled MicL/peptide system was evaluated using the BioLector™ system for MTP cultivations (Figs. 4, 5). Lpp was downregulated for 6.3 generations, as opposed to merely 1.3 induced generations with the coupled system. When MicL was expressed via the multicopy plasmid pMicL^MC^(ara), knockout-like conditions were achieved (Fig. 5B). To the contrary, Lpp was knocked down to a lower extent with the single copy plasmid pMicL^SC^(ara). These results are in accordance with our flow cytometry results, where SYTOX™ green uptake was used as permeability indicator (Figs. 5A, 7A). The reference cultivations showed an unaltered permeability, while cells that expressed MicL showed a distinct shift of the peak to the right, indicating an increased permeability. This effect was again more distinct when MicL was expressed via the multicopy plasmid. In view of the lower gene dosage with the single copy plasmid, the high Lpp mRNA transcription rate was not compensated (Nakamura and Inouye 1979), and a comparatively high amount of Lpp was still present in the cells. These findings are also in accordance with cultivations that utilized the coupled system, as discussed before. Interestingly, Lpp downregulation resulted in higher total specific peptide titers (Fig. 4D–F), especially when MicL was expressed from pMicL^MC^(ara). Lpp is numerically the most abundant protein in *E. coli*, and its expression occupies > 5% of all ribosomes (Li et al. 2014). We suggest that increased peptide expression levels are a result of newly freed metabolic resources alongside access to otherwise occupied ribosomes.

The decoupled MicL/peptide system was evaluated regarding extracellular peptide production in stirred-tank reactors under production conditions in fed-batch mode (Figs. 6, 7). MicL was induced by LAra addition during batch phase, while IPTG was added during fed-batch phase. Lpp was downregulated to a great extent after only 2 h of induction; however, this was accompanied by severe growth restrictions. In contrast, when MicL was induced during MTP cultivations during the batch phase, the cells only showed reduced growth rates during later stages of cultivation although knockout-like conditions were achieved (Figs. 4, 5). This is unlikely to be a result of the induction conditions, as the specific LAra concentrations were four times higher during MTP cultivations. We therefore believe this to be a consequence of the media composition, as the medium for MTP cultivations was designed for lower cell densities, resulting in lower osmolarity. Given that the osmolarity of the medium can negatively impact the growth rate (Record et al. 1998; Pilizota and Shaevitz 2014), a compromised OM might even further affect the susceptibility to osmotic stress. Moreover, thiamin was supplemented to the medium during MTP cultivations, thereby possibly compensating the reduced growth rates upon Lpp knockdown (Lakaye et al. 2004). The specific total peptide titer was increased compared to reference cultivation; however, due to the lower biomass of Lpp knockdown strains, the volumetric titers were also lower. Although Lpp was strongly downregulated, the extracellular peptide content did not increase substantially. This is surprising in view of the strong increase in extracellular peptide during MTP cultivations. The pH steadily decreases over time during MTP cultivations due to insufficient buffering of the system upon acetate production (Toeroek et al. 2015). It was shown before that the pH influences the OM composition, thus potentially playing an important role for the OM permeability (Sato et al. 2000). In consequence, this suggests that to fully exploit the potential of a reduced Lpp content during stirred-tank reactor cultivations, additional factors like the pH, medium composition, and growth rate must be considered.

When performing RP-HPLC with samples that were derived from Lpp-downregulating cells during STR cultivations, almost no extracellular peptide could be identified. This contrasted results from SDS-PAGE analysis, where bands ascribed to CASPON-SST were clearly visible (Figure S3). More so, these findings contradict results from MTP cultivations, where extracellular peptide could clearly be identified via RP-HPLC and reflected SDS-PAGE results. We therefore concluded that the extracellular recombinant CASPON-SST could be enclosed inside OMVs, subsequently complicating accurate quantification. During both MTP and STR cultivations with Lpp-downregulating cells, we found evidence for the formation of OMVs (Figs. 8, 9). When Lpp was downregulated, the OM appeared uneven and wavy, as opposed to the smooth OM from the reference, which complements previously published results (Mathelié-Guinlet et al. 2020). Surprisingly, the cells found in the reference cultivation were spherical and not rod-shaped. Spherical *E. coli* cells were reported before and were a result of certain gene knockouts (Govindarajan et al. 2013; Lu et al. 2022; Fivenson et al. 2023). We therefore assume that the Δ*araABCD* background in the reference production strain caused the spherical shape. This was likely to be restored to rod shapes in the plasmid-carrying strains by the presence of the *araC* gene on the plasmid and the positive regulation upon LAra addition. Genes activated by AraC in turn could potentially contribute to the structural features of *E. coli* (Stringer et al. 2014), especially during C-limited conditions in bioreactors. OMVs were found excessively when MicL was expressed from pMicL^MC^(ara). Intriguingly, while peptides were readily extracted via HCl from OMVs derived by MTP cultures, the same process was not successful with OMVs from STR cultures. This discrepancy may arise from variations in magnesium and calcium concentrations across the different media. Mg^2+^ and Ca^2+^ are important components for *E. coli* and positively influence the OM integrity by binding to LPS (Jeworrek et al. 2011; Herrmann et al. 2015; Warsi et al. 2018). The specific Mg^2+^ and Ca^2+^ concentrations were 6- and 115-fold higher, respectively, during STR cultivations compared to MTP cultivations. This suggests that OMVs from MTP cultures were less stable compared to OMVs from STR cultures. Moreover, the Mg^2+^ and Ca^2+^ ions do not only stabilize the OMVs, but the OM in general, which would explain why the OM permeability was increased during STR cultivations, but peptide leakage was only poorly enhanced. The importance of Mg^2+^ and Ca^2+^ ions in combination with leaky *E. coli* hosts was subject to previously patented systems involving *lpp* deletion strains, complementing our findings (Dassler et al. 2008; Dassler and Kujau 2021). Moreover, Fu et al. showed that the deletion of *lpp* resulted in a higher OM permeability compared to *pal* deletion. The secretion of different proteins and an antimicrobial peptide, however, was worse compared to *pal* deletion (Fu et al. 2022). These observations match our results and show that the uptake of fluorescent probe molecules (permeability) does not necessarily reflect the actual capability of a system to release peptides or proteins (leakiness). Consequently, permeability must be distinguished from leakiness and cannot be used interchangeably.

Finally, our results suggest that low Lpp levels in the cells alone are not responsible for the leakage of periplasmic contents (permeability vs. leakiness) and OMV formation. Rather, we believe that several influencing factors interact and must be harmonized in future work to reliably promote the release of periplasmic proteins. Leaky *E. coli* strains with mutations or deletions in the *lpp* gene have been used for recombinant protein production for many years. A variety of proteins and peptides were thus far successfully secreted up to 94% from their host. Among others, these include penicillin G acylase (Orr et al. 2012), streptavidin (Müller et al. 2016), human parathyroid hormone 1–84 (Chen et al. 2014), and L-carnitine (Ni et al. 2007). Here, we describe for the first time the use of MicL for inducible downregulation of Lpp during fed-batch processes for recombinant peptide production in bioreactors. Depending on the system and cultivation strategy, we were able to achieve the release of 80–100% of recombinant CASPON-SST into the cultivation medium. Many of the previous studies utilizing leaky *E. coli* hosts were carried out for research purposes at small scale in shake flasks. With the data we present here, we propose the inducible MicL system for in-process Lpp knockdown as valuable alternative to *lpp* knockout.

We were able to achieve high extracellular peptide titers with the inducible MicL system for Lpp knockdown as alternative to *lpp* knockout. Nonetheless, it needs to be emphasized that further optimization is needed of the individual approaches. Especially for industrial applications, individual process parameters need to be thoroughly investigated to elucidate their impact on peptide expression, permeability, leakiness, and the viability of the culture. Furthermore, we believe that our system should not be limited to the release of intracellular proteins/peptides into the cultivation medium. Instead, we suggest that a flexible system for OM permeability could find use in the generation of OMVs as promising biotechnological product. In this study, we did not originally intend to favor the generation of OMVs, and the purification and analysis thereof were beyond the scope of this work. However, due to the great potential of OMVs for biotechnological and therapeutic applications, we will investigate OMV formation, especially regarding scalability and purity, in the future. Lastly, inducible permeability might be a valuable strategy when the uptake of certain media components needs to be increased, and leakiness is not a requirement. The permeabilization of host strains for facilitating the release of recombinant POIs to the medium is of great interest for both industry and research. Considering the impact of Lpp on production hosts and potential applications of inducible permeability, we are confident that our results are a valuable contribution to future efforts regarding extracellular protein and peptide production with *E. coli*.

## Supplementary Information

Below is the link to the electronic supplementary material.

Supplementary file1 (PDF 2377 KB)

## Data Availability

All data supporting the findings of this study are available within the paper and its Supplementary Information.

## Declarations

This article does not contain any studies with animals performed by any of the authors.
