# Supplementary material for: Extracellular peptide production in Escherichia coli by inducible downregulation of lipoprotein Lpp via MicL sRNA
Source: Appl Microbiol Biotechnol. 2025 Jun 4;109(1):136. doi: 10.1007/s00253-025-13524-z (PMC12137417; doi:10.1007/s00253-025-13524-z)
Supplement: Supplementary file 1 — Supplementary file1 (PDF 2377 KB) [file 253_2025_13524_MOESM1_ESM.pdf]

Extracellular peptide production in *Escherichia coli* by inducible downregulation of lipoprotein Lpp via MicL sRNA

Martin Gibisch[1], Pawel Gorecki[1], Christopher Tauer[1], Esther Egger[1], Matthias Müller[1], Bernd Albrecht[2], Rainer Hahn[1], Gerald Striedner[1], Monika Cserjan-Puschmann[1,*],

Affiliations:

1) Christian Doppler Laboratory for production of next-level biopharmaceuticals in *E. coli*, Institute of Bioprocess Science and Engineering, BOKU University, Muthgasse 18, 1190, Vienna, Austria

2) Boehringer-Ingelheim RCV GmbH & Co KG, Doktor-Boehringer-Gasse 5-11, 1120, Vienna, Austria

*: Corresponding Author

Supplementary material

Table S1: RT-qPCR primers and sequences used in this study.

Primer	Sequence
16S RNA forward	CCTCATAAAGTGCGTCGTAGT
16S RNA reverse	CTGATTCACCGTGGCATTCT
CASPON-SST forward	CGCAACAAAGAGCGAAAGG
CASPON-SST reverse	CATCGCTGGGTTTGAGTTTG
Lpp forward	CGGTAATCCTGGGTTCTACTC
Lpp reverse	TTCACGTCGTTGCTCAGC

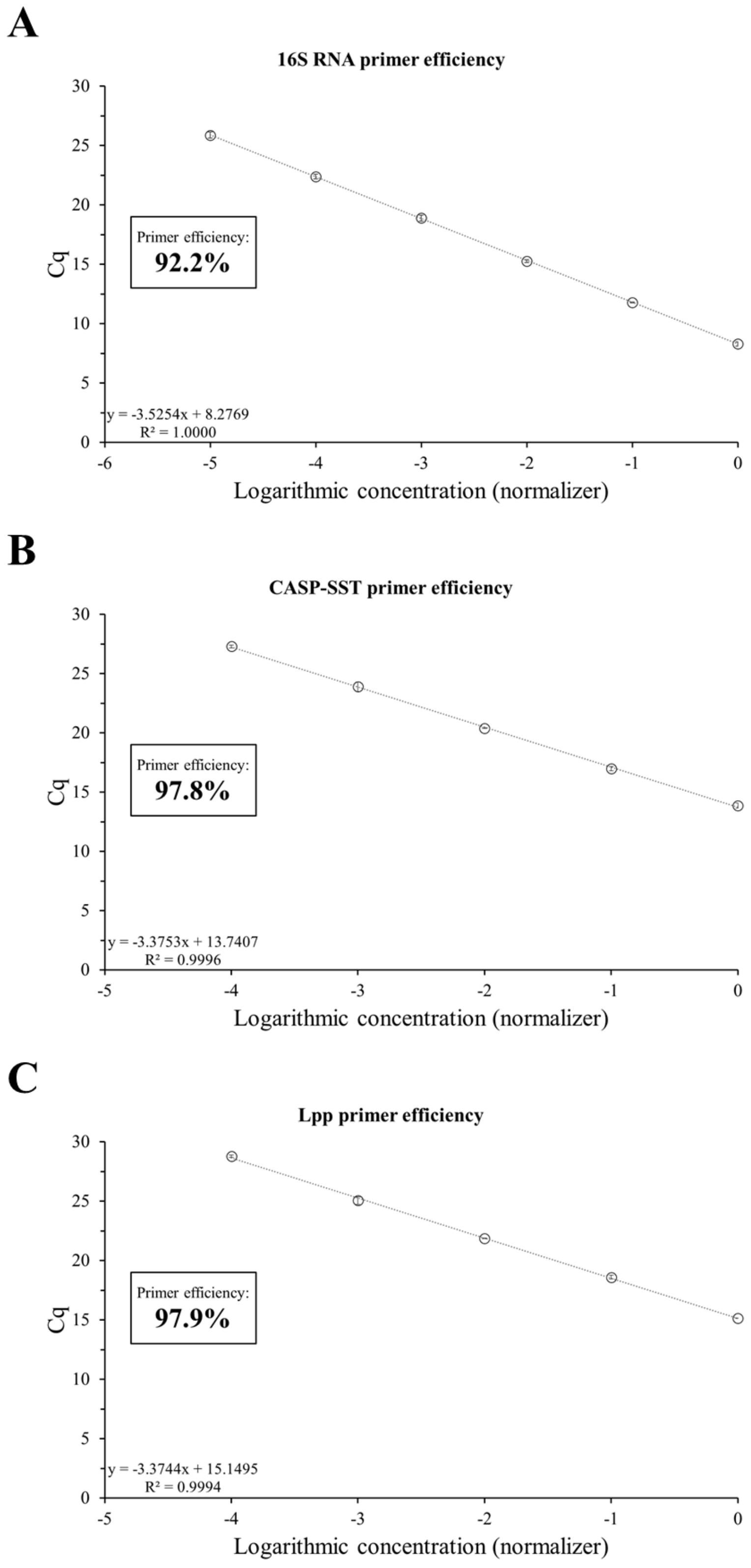

Figure S1: Primer efficiency standard curves for 16S RNA (**A**), CASPON-SST (**B**), and Lpp (**C**). Cq values derived from technical triplicates. Error bars represent the maximal and minimal value.

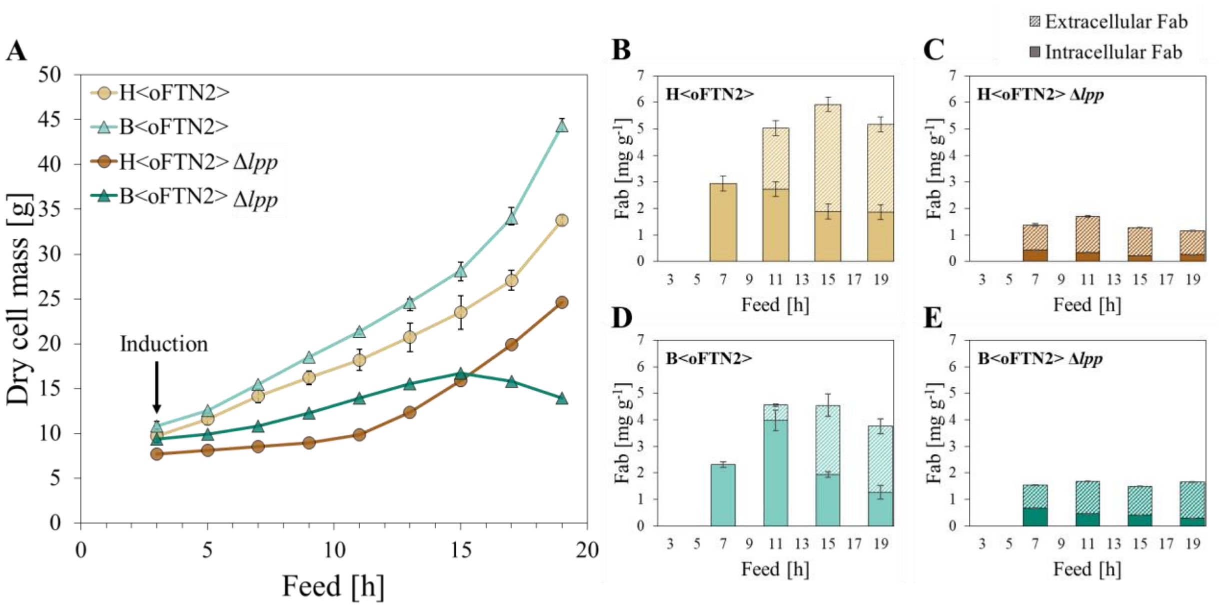

Figure S2: Production of the Fab fragment FTN2 in HMS174(DE3)- and BL21(DE3)-derived host strains with and without Δ*lpp* background. **A**) growth curves, **B, C, D, E**) specific FTN2 content throughout the cultivation determined by ELISA. The cassette for FTN2 expression was integrated into the genome of the host strains and is indicated as "<>". The OmpA signal sequence (abbreviated as "o") was used for translocation into the periplasm. Error bars represent biological duplicates in case of growth curves, and technical triplicates in case of specific Fab content.

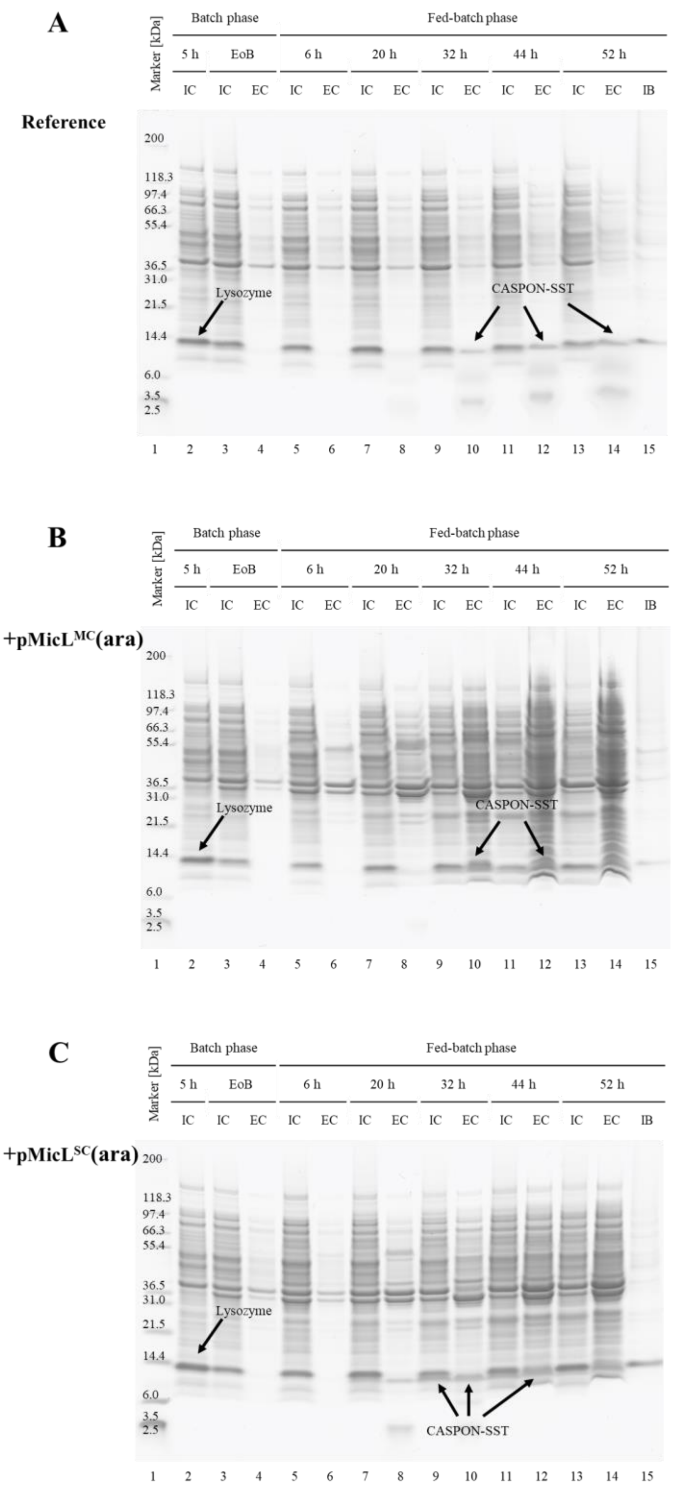

Figure S3: SDS-PAGE analysis of intracellular (IC) and extracellular (EC) fractions during bioreactor cultivations with strains expressing the peptide CASPON-SST and MicL sRNA. **A**) Reference cultivation of BΔ*ara*<oCASPON-SST> (Reference) induced with L-Arabinose, **B**) cultivation of BΔ*ara*<oCASPON-SST> pMicL^{MC}(ara), **C**) cultivation of BΔ*ara*<oCASPON-SST> pMicL^{SC}(ara). Intracellular samples contain lysozyme (~14 kDa) for enzymatic cell lysis, indicated by a black arrow in lane 2 of each gel. CASPON-SST bands are present below lysozyme bands at ~12 kDa and are also indicated by black arrows. EoB: end of batch.

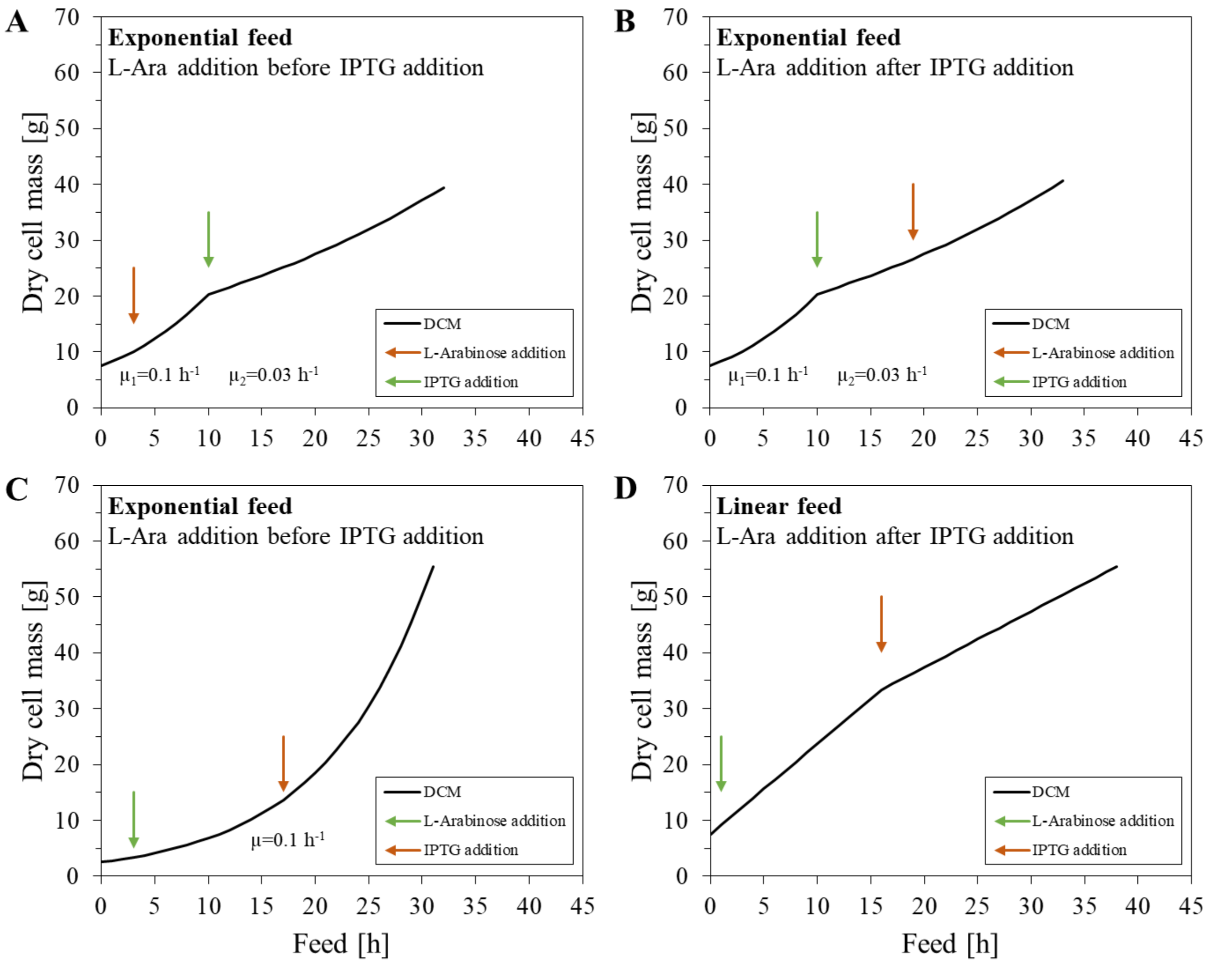

Figure S4: Cultivation strategies for Lpp downregulation and peptide expression in stirred tank bioreactors. Addition of L-Arabinose or IPTG is indicated as green and orange arrow, respectively. The dry cell mass (DCM) was calculated according to the respective specific growth rates (exponential) or the feed rate (linear). The processes were designed according to the number of induced generations with either L-Arabinose or IPTG, and the induction strength.

Accession	-10lgP	Coverage (%)	Coverage (%) Gel1_Band1	Area Gel1_Band1	#Peptides	#Unique	#Spec Gel1_Band1	PTM	Avg. Mass	Description
A0A140NEY2	531.20	90	90	5.7229E9	195	195	571	Y	49912	Maltoporin OS=Escherichia coli (strain B / BL21-DE3) OX=469008 GN=lamB PE=2 SV=1
A0A140NAN5	430.55	86	86	1.1655E9	100	97	185	Y	39333	Porin Gram-negative type OS=Escherichia coli (strain B / BL21-DE3) OX=469008 GN=ECBD_2666 PE=3 SV=1
A0A140ND02	396.04	71	71	1.5596E8	65	65	98	Y	62656	Extracellular solute-binding protein family 5 OS=Escherichia coli (strain B / BL21-DE3) OX=469008 GN=ECBD_2379 PE=3 SV=1
A0A140SS73	391.60	81	81	1.7971E8	62	62	91	Y	56231	Glycerol kinase OS=Escherichia coli (strain B / BL21-DE3) OX=469008 GN=glpK PE=3 SV=1
A0A140N510	361.08	75	75	6.6262E7	42	42	65	Y	59644	Phosphoenolpyruvate carboxykinase (ATP) OS=Escherichia coli (strain B / BL21-DE3) OX=469008 GN=pckA PE=3 SV=1
A0A140N3Z1	355.14	71	71	6.2952E7	37	37	58	Y	55527	Peptidase M16 domain protein OS=Escherichia coli (strain B / BL21-DE3) OX=469008 GN=ECBD_0212 PE=3 SV=1
A0A140NBF1	345.28	62	62	3.8144E7	39	39	52	Y	57913	Glucans biosynthesis protein G OS=Escherichia coli (strain B / BL21-DE3) OX=469008 GN=mdoG PE=3 SV=1
A0A140N9Z6	330.23	59	59	3.2365E7	31	31	45	Y	52224	Aldehyde Dehydrogenase OS=Escherichia coli (strain B / BL21-DE3) OX=469008 GN=ECBD_2225 PE=3 SV=1
A0A140N3X6	327.58	73	73	3.3926E7	41	41	56	Y	60294	Extracellular solute-binding protein family 5 OS=Escherichia coli (strain B / BL21-DE3) OX=469008 GN=ECBD_0192 PE=3 SV=1
A0A140NCL5	327.35	60	60	1.4035E7	25	25	36	Y	63637	Periplasmic trehalase OS=Escherichia coli (strain B / BL21-DE3) OX=469008 GN=treA PE=3 SV=1
A0A140NBS3	325.01	62	62	5.4805E7	37	37	50	Y	50689	Dihydrolipoyl dehydrogenase OS=Escherichia coli (strain B / BL21-DE3) OX=469008 GN=ECBD_3503 PE=3 SV=1
A0A140N7U4	321.57	64	64	2.8466E7	28	28	41	Y	51496	6-phosphogluconate dehydrogenase, decarboxylating OS=Escherichia coli (strain B / BL21-DE3) OX=469008 GN=ECBD_1630 PE=3 SV=1
A0A140N5A7	320.87	57	57	2.2243E7	28	28	35	Y	60694	Extracellular solute-binding protein family 5 OS=Escherichia coli (strain B / BL21-DE3) OX=469008 GN=ECBD_0719 PE=4 SV=1
A0A140SS76	316.41	70	70	1.7086E7	30	30	40	Y	57329	Bifunctional purine biosynthesis protein PurH OS=Escherichia coli (strain B / BL21-DE3) OX=469008 GN=purH PE=3 SV=1
A0A140NH65	311.66	65	65	2.8093E7	30	30	53	Y	57329	60 kDa chaperonin OS=Escherichia coli (strain B / BL21-DE3) OX=469008 GN=groL PE=3 SV=1
A0A140N8Y0	309.39	57	57	9.0781E6	27	27	31	Y	60299	Fumarate hydratase class I OS=Escherichia coli (strain B / BL21-DE3) OX=469008 GN=ECBD_2034 PE=3 SV=1
A0A140NAC5	305.43	71	71	1.6162E7	32	32	35	Y	59900	Extracellular solute-binding protein family 5 OS=Escherichia coli (strain B / BL21-DE3) OX=469008 GN=ECBD_2287 PE=3 SV=1
A0A140NCM7	304.62	63	63	1.6355E7	32	32	44	Y	60824	5'-Nucleotidase domain protein OS=Escherichia coli (strain B / BL21-DE3) OX=469008 GN=ECBD_3176 PE=3 SV=1
A0A140N984	288.73	60	60	3.2603E7	34	34	45	Y	57668	Extracellular solute-binding protein family 5 OS=Escherichia coli (strain B / BL21-DE3) OX=469008 GN=ECBD_2152 PE=3 SV=1
A0A140N9Y8	287.65	65	65	1.4539E7	30	30	48	Y	61308	Glucans biosynthesis protein D OS=Escherichia coli (strain B / BL21-DE3) OX=469008 GN=mdoD PE=3 SV=1

Figure S5: Mass spectrometry results of gel band derived from SDS-PAGE. The protein identified is most likely to be maltoporin (LamB).

Accession	-10lgP	Coverage (%)	Coverage (%) Gel1_Band2	Area Gel1_Band2	#Peptides	#Unique	#Spec Gel1_Band2	PTM	Avg. Mass	Description
A0A140N3X6	352.66	80	80	6.7401E8	93	93	197	Y	60294	Extracellular solute-binding protein family 5 OS=Escherichia coli (strain B / BL21-DE3) OX=469008 GN=ECBD_0192 PE=3 SV=1
A0A140NAN5	290.31	71	71	2.9427E8	45	44	77	Y	39333	Porin Gram-negative type OS=Escherichia coli (strain B / BL21-DE3) OX=469008 GN=ECBD_2666 PE=3 SV=1
A0A140N6A8	275.58	72	72	7.595E7	46	46	68	Y	53741	Type I secretion outer membrane protein, TolC family OS=Escherichia coli (strain B / BL21-DE3) OX=469008 GN=ECBD_0704 PE=3 SV=1
A0A140NI72	265.24	63	63	4.9126E7	42	42	67	Y	54069	Ketol-acid reductoisomerase (NADP(+)) OS=Escherichia coli (strain B / BL21-DE3) OX=469008 GN=ilvC PE=3 SV=1
A0A140N7U4	230.88	54	54	1.1898E7	25	25	35	Y	51496	6-phosphogluconate dehydrogenase, decarboxylating OS=Escherichia coli (strain B / BL21-DE3) OX=469008 GN=ECBD_1630 PE=3 SV=1
A0A140NAP3	227.15	66	66	2.4998E7	24	24	43	Y	35541	L-arabinose-binding periplasmic protein OS=Escherichia coli (strain B / BL21-DE3) OX=469008 GN=ECBD_1740 PE=3 SV=1
A0A140N9Y8	213.85	47	47	9.6237E6	27	27	38	Y	61308	Glucans biosynthesis protein D OS=Escherichia coli (strain B / BL21-DE3) OX=469008 GN=mdoD PE=3 SV=1
A0A140ND72	203.88	42	42	7.2551E6	19	19	25	Y	55222	ATP synthase subunit alpha OS=Escherichia coli (strain B / BL21-DE3) OX=469008 GN=atpA PE=3 SV=1
A0A140N9Z6	203.47	39	39	4.3018E6	17	17	23	Y	52224	Aldehyde Dehydrogenase OS=Escherichia coli (strain B / BL21-DE3) OX=469008 GN=ECBD_2225 PE=3 SV=1
A0A140NAV6	199.53	40	40	2.9354E6	19	19	25	Y	49354	Periplasmic serine endoprotease DegP-like OS=Escherichia coli (strain B / BL21-DE3) OX=469008 GN=ECBD_3457 PE=3 SV=1
A0A140N3Z1	198.88	36	36	5.5434E6	17	17	23	Y	55527	Peptidase M16 domain protein OS=Escherichia coli (strain B / BL21-DE3) OX=469008 GN=ECBD_0212 PE=3 SV=1
A0A140NC34	183.98	41	41	1.7301E7	23	23	32	Y	56470	Extracellular solute-binding protein family 5 OS=Escherichia coli (strain B / BL21-DE3) OX=469008 GN=ECBD_2793 PE=3 SV=1
A0A140NET2	182.09	49	49	1.5099E7	26	26	44	Y	48193	Trigger factor OS=Escherichia coli (strain B / BL21-DE3) OX=469008 GN=tig PE=3 SV=1
A0A140N6W0	176.03	43	43	5.4082E6	14	14	20	Y	43284	Elongation factor Tu OS=Escherichia coli (strain B / BL21-DE3) OX=469008 GN=tuf PE=1 SV=1
A0A140NCI6	176.03	43	43	5.4082E6	14	14	20	Y	43314	Elongation factor Tu OS=Escherichia coli (strain B / BL21-DE3) OX=469008 GN=tuf PE=3 SV=1
A0A140N5T5	175.37	28	28	3.1877E6	14	14	20	Y	47205	Periplasmic serine endoprotease DegP-like OS=Escherichia coli (strain B / BL21-DE3) OX=469008 GN=ECBD_0513 PE=3 SV=1
A0A140NAN3	159.61	33	33	1.3471E6	14	14	15	Y	45770	Isocitrate dehydrogenase [NADP] OS=Escherichia coli (strain B / BL21-DE3) OX=469008 GN=ECBD_2463 PE=4 SV=1
A0A140N460	154.92	40	40	3.4251E6	17	17	24	Y	51858	Cell division protein FtsP OS=Escherichia coli (strain B / BL21-DE3) OX=469008 GN=ftsP PE=3 SV=1
A0A140N7B7	150.35	24	24	8.5951E5	9	9	13	Y	52022	Inosine-5'-monophosphate dehydrogenase OS=Escherichia coli (strain B / BL21-DE3) OX=469008 GN=guaB PE=3 SV=1
A0A140NHS0	148.43	48	48	2.3843E6	17	17	28	Y	50325	ATP synthase subunit beta OS=Escherichia coli (strain B / BL21-DE3) OX=469008 GN=atpD PE=3 SV=1
A0A140NAR3	146.12	38	38	2.4876E6	18	18	24	Y	52184	Cysteine--tRNA ligase OS=Escherichia coli (strain B / BL21-DE3) OX=469008 GN=cysS PE=3 SV=1
A0A140NGN0	145.62	26	26	1.5561E6	11	11	12	Y	52356	Aspartate ammonia-lyase OS=Escherichia coli (strain B / BL21-DE3) OX=469008 GN=ECBD_3892 PE=3 SV=1
A0A140NG49	144.69	32	32	1.2554E7	15	15	23	Y	56583	Multicopper oxidase type 3 OS=Escherichia coli (strain B / BL21-DE3) OX=469008 GN=ECBD_3496 PE=4 SV=1
A0A140SSA2	144.00	28	28	2.6096E6	15	15	20	Y	51904	Glutamine synthetase OS=Escherichia coli (strain B / BL21-DE3) OX=469008 GN=ECBD_4157 PE=3 SV=1

Figure S6: Mass spectrometry results of gel band derived from SDS-PAGE. The protein was identified to be the extracellular solute binding protein family 5.

Accession	-10lgP	Coverage (%)	Coverage (%) Gel1 Band 2	Area Gel1 Band 2	#Peptides	#Unique	#Spec Gel1 Band 2	PTM	Avg. Mass	Description
A0A140NCD0	462.95	82	82	5.2676E8	84	84	191	Y	43388	Maltodextrin-binding protein OS=Escherichia coli (strain B / BL21-DE3) OX=469008 GN=ECBD_4002 PE=1 SV=1
A0A140N8E1	414.06	89	89	1.3564E8	52	52	106	Y	41118	Phosphoglycerate kinase OS=Escherichia coli (strain B / BL21-DE3) OX=469008 GN=pgk PE=3 SV=1
A0A140ND68	397.43	87	87	1.6604E8	55	54	95	Y	43573	Aminotransferase OS=Escherichia coli (strain B / BL21-DE3) OX=469008 GN=ECBD_2667 PE=3 SV=1
A0A140N821	384.96	80	80	6.56E7	36	36	51	Y	39147	Fructose-bisphosphate aldolase OS=Escherichia coli (strain B / BL21-DE3) OX=469008 GN=ECBD_0813 PE=3 SV=1
A0A140N2E4	370.90	73	73	3.8106E7	34	29	49	Y	39076	Extracellular ligand-binding receptor OS=Escherichia coli (strain B / BL21-DE3) OX=469008 GN=ECBD_0281 PE=3 SV=1
A0A140NBF4	367.59	72	72	1.3062E8	42	42	69	Y	41393	Succinate--CoA ligase [ADP-forming] subunit beta OS=Escherichia coli (strain B / BL21-DE3) OX=469008 GN=sucC PE=3 SV=1
A0A140N4Y5	362.02	70	70	3.0658E7	29	29	44	Y	40018	Aspartate-semialdehyde dehydrogenase OS=Escherichia coli (strain B / BL21-DE3) OX=469008 GN=asd PE=3 SV=1
A0A140N627	327.45	69	69	1.5037E7	22	22	35	Y	41952	S-adenosylmethionine synthase OS=Escherichia coli (strain B / BL21-DE3) OX=469008 GN=metK PE=3 SV=1
A0A140N9N7	326.49	64	64	1.678E7	23	23	32	Y	41650	UDP-4-amino-4-deoxy-L-arabinose--oxoglutarate aminotransferase OS=Escherichia coli (strain B / BL21-DE3) OX=469008 GN=arnB
A0A140NDI4	323.05	72	72	4.0762E7	29	29	59	Y	39840	Phosphoserine aminotransferase OS=Escherichia coli (strain B / BL21-DE3) OX=469008 GN=serC PE=3 SV=1
A0A140N7M5	314.99	60	60	8.47E6	17	17	18	Y	43290	Acetate kinase OS=Escherichia coli (strain B / BL21-DE3) OX=469008 GN=ackA PE=3 SV=1
A0A140NC10	311.29	63	63	2.2689E7	26	26	37	Y	48015	Citrate synthase OS=Escherichia coli (strain B / BL21-DE3) OX=469008 GN=ECBD_2941 PE=3 SV=1
A0A140NBW7	307.71	72	72	1.7908E7	25	25	42	Y	39517	3-isopropylmalate dehydrogenase OS=Escherichia coli (strain B / BL21-DE3) OX=469008 GN=leuB PE=3 SV=1
A0A140NBZ6	307.44	68	68	1.1346E7	21	21	25	Y	42295	Extracellular solute-binding protein family 1 OS=Escherichia coli (strain B / BL21-DE3) OX=469008 GN=ECBD_2199 PE=3 SV=1
A0A140NC97	307.37	83	83	6.6786E7	30	30	49	Y	20761	Alkyl hydroperoxide reductase C OS=Escherichia coli (strain B / BL21-DE3) OX=469008 GN=ECBD_3047 PE=1 SV=1
A0A140N6W0	306.56	68	68	1.3744E7	21	21	27	Y	43284	Elongation factor Tu OS=Escherichia coli (strain B / BL21-DE3) OX=469008 GN=tuf PE=1 SV=1
A0A140NCI6	306.56	68	68	1.3744E7	21	21	27	Y	43314	Elongation factor Tu OS=Escherichia coli (strain B / BL21-DE3) OX=469008 GN=tuf PE=3 SV=1
A0A140NFM6	300.97	61	61	3.6879E6	18	18	22	Y	40324	Cell division protein FtsZ OS=Escherichia coli (strain B / BL21-DE3) OX=469008 GN=ftsZ PE=3 SV=1
A0A140N5L9	296.60	76	76	5.6614E6	16	16	21	Y	34490	Cysteine synthase OS=Escherichia coli (strain B / BL21-DE3) OX=469008 GN=ECBD_1267 PE=3 SV=1
A0A140NEW5	295.18	59	59	1.2935E7	20	19	29	Y	43522	Aminotransferase OS=Escherichia coli (strain B / BL21-DE3) OX=469008 GN=ECBD_3979 PE=3 SV=1
A0A140N6E5	284.23	66	66	8.7971E6	20	20	25	Y	47014	D-tagatose-1,6-bisphosphate aldolase subunit GatZ OS=Escherichia coli (strain B / BL21-DE3) OX=469008 GN=gatZ PE=3 SV=1
A0A140N487	280.30	54	54	5.401E6	22	22	25	Y	43045	2-amino-3-ketobutyrate coenzyme A ligase OS=Escherichia coli (strain B / BL21-DE3) OX=469008 GN=kbl PE=3 SV=1
A0A140NBT6	280.21	65	65	5.8938E6	18	18	23	Y	38188	Major capsid protein E OS=Escherichia coli (strain B / BL21-DE3) OX=469008 GN=ECBD_2868 PE=3 SV=1
A0A140NB56	280.04	56	56	4.1234E6	18	18	19	Y	45694	Glucose-1-phosphatase OS=Escherichia coli (strain B / BL21-DE3) OX=469008 GN=ECBD_2592 PE=4 SV=1
A0A140N6V3	273.59	57	57	2.9745E6	16	16	18	Y	39502	NADH:flavin oxidoreductase/NADH oxidase OS=Escherichia coli (strain B / BL21-DE3) OX=469008 GN=ECBD_1993 PE=4 SV=1
A0A140N783	268.21	73	73	2.3821E7	24	24	36	Y	35533	Glyceraldehyde-3-phosphate dehydrogenase OS=Escherichia coli (strain B / BL21-DE3) OX=469008 GN=ECBD_1865 PE=3 SV=1
A0A140N7B0	267.56	67	67	5.9274E6	20	19	26	Y	43767	Acetylornithine/succinyldiaminopimelate aminotransferase OS=Escherichia coli (strain B / BL21-DE3) OX=469008 GN=argD PE=3 SV=
A0A140N2U0	266.78	63	63	1.0078E7	21	21	28	Y	36512	DNA-directed RNA polymerase subunit alpha OS=Escherichia coli (strain B / BL21-DE3) OX=469008 GN=rpoA PE=3 SV=1
A0A140NFT4	266.20	46	46	4.1163E6	13	13	13	Y	41431	Carbamoyl-phosphate synthase small chain OS=Escherichia coli (strain B / BL21-DE3) OX=469008 GN=carA PE=3 SV=1
OmpF	260.66	60	60	6.668E6	19	19	21	Y	39333	OmpF

Figure S7: Mass spectrometry results of gel band derived from SDS-PAGE. The protein was identified as the maltose binding protein (MBP).

Accession	-10lgP	Coverage (%)	Coverage (%) Gel2 Band 2	Area Gel2 Band 2	#Peptides	#Unique	#Spec Gel2 Band 2	PTM	Avg. Mass	Description
A0A140NAN5	428.64	74	74	1.0362E9	67	67	281	Y	39333	Porin Gram-negative type OS=Escherichia coli (strain B / BL21-DE3) OX=469008 GN=ECBD_2666 PE=3 SV=1
OmpF	428.64	74	74	1.0362E9	67	67	281	Y	39333	OmpF
A0A140N2E4	382.73	63	63	1.4546E8	36	34	82	Y	39076	Extracellular ligand-binding receptor OS=Escherichia coli (strain B / BL21-DE3) OX=469008 GN=ECBD_0281 PE=3 SV=1
A0A140N5L9	344.80	78	78	8.1187E7	24	24	68	Y	34490	Cysteine synthase OS=Escherichia coli (strain B / BL21-DE3) OX=469008 GN=ECBD_1267 PE=3 SV=1
A0A140NAP3	325.38	60	60	9.873E7	22	22	96	Y	35541	L-arabinose-binding periplasmic protein OS=Escherichia coli (strain B / BL21-DE3) OX=469008 GN=ECBD_1740 PE=3 SV=1
A0A140N802	316.22	61	61	4.3331E7	21	21	30	Y	40833	Glycerophosphoryl diester phosphodiesterase OS=Escherichia coli (strain B / BL21-DE3) OX=469008 GN=ECBD_1421 PE=4 SV=1
A0A140N4Y5	312.42	65	65	2.0308E7	22	22	29	Y	40018	Aspartate-semialdehyde dehydrogenase OS=Escherichia coli (strain B / BL21-DE3) OX=469008 GN=asd PE=3 SV=1
A0A140NDI4	277.13	55	55	5.8436E6	16	16	21	Y	39840	Phosphoserine aminotransferase OS=Escherichia coli (strain B / BL21-DE3) OX=469008 GN=serC PE=3 SV=1
A0A140N783	270.72	67	67	2.8655E7	21	20	37	Y	35533	Glyceraldehyde-3-phosphate dehydrogenase OS=Escherichia coli (strain B / BL21-DE3) OX=469008 GN=ECBD_1865 PE=3 SV=1
A0A140N6J5	270.54	53	53	7.8806E6	14	12	20	Y	39379	Extracellular ligand-binding receptor OS=Escherichia coli (strain B / BL21-DE3) OX=469008 GN=ECBD_0283 PE=3 SV=1
A0A140N4S8	261.29	77	77	6.2154E6	16	16	19	Y	36842	Outer membrane protein assembly factor BamC OS=Escherichia coli (strain B / BL21-DE3) OX=469008 GN=bamC PE=3 SV=1
A0A140NGD3	258.49	55	55	2.9415E6	17	17	19	N	37024	Phosphate-binding protein PstS OS=Escherichia coli (strain B / BL21-DE3) OX=469008 GN=ECBD_4304 PE=3 SV=1
A0A140N6W0	252.37	39	39	5.057E6	13	13	15	Y	43284	Elongation factor Tu OS=Escherichia coli (strain B / BL21-DE3) OX=469008 GN=tuf PE=1 SV=1
A0A140NCI6	252.37	39	39	5.057E6	13	13	15	Y	43314	Elongation factor Tu OS=Escherichia coli (strain B / BL21-DE3) OX=469008 GN=tuf PE=3 SV=1
A0A140N775	248.08	66	66	5.3231E6	14	14	16	Y	32337	Malate dehydrogenase OS=Escherichia coli (strain B / BL21-DE3) OX=469008 GN=mdh PE=3 SV=1
A0A140N9C2	242.62	56	56	2.0494E6	17	17	21	Y	37628	Sulfate ABC transporter, periplasmic sulfate-binding protein OS=Escherichia coli (strain B / BL21-DE3) OX=469008 GN=ECBD_1256 PE
A0A140NBF2	219.54	42	42	1.9636E6	9	9	11	Y	35048	EIIAB-Man OS=Escherichia coli (strain B / BL21-DE3) OX=469008 GN=ECBD_1824 PE=4 SV=1
A0A140N2U0	217.94	53	53	5.4176E6	11	11	14	Y	36512	DNA-directed RNA polymerase subunit alpha OS=Escherichia coli (strain B / BL21-DE3) OX=469008 GN=rpoA PE=3 SV=1
A0A140ND68	215.25	34	34	8.7024E5	12	12	14	Y	43573	Aminotransferase OS=Escherichia coli (strain B / BL21-DE3) OX=469008 GN=ECBD_2667 PE=3 SV=1
A0A140N6C8	213.21	39	39	2.7293E6	10	10	10	N	40411	Membrane-bound lytic murein transglycosylase A OS=Escherichia coli (strain B / BL21-DE3) OX=469008 GN=ECBD_0909 PE=4 SV=1
A0A140N9N8	211.30	34	34	1.9636E6	8	8	8	Y	40826	Putrescine-binding periplasmic protein OS=Escherichia coli (strain B / BL21-DE3) OX=469008 GN=ECBD_2740 PE=3 SV=1
A0A140N7C1	205.24	43	43	3.1459E6	12	12	13	Y	37438	Tryptophan--tRNA ligase OS=Escherichia coli (strain B / BL21-DE3) OX=469008 GN=trpS PE=3 SV=1
A0A140N6N0	203.11	39	39	1.4848E6	10	10	10	Y	36832	Phenylalanine--tRNA ligase alpha subunit OS=Escherichia coli (strain B / BL21-DE3) OX=469008 GN=pheS PE=3 SV=1
A0A140SS92	202.82	50	50	2.2428E6	11	11	11	N	36659	Sulfate ABC transporter, periplasmic sulfate-binding protein OS=Escherichia coli (strain B / BL21-DE3) OX=469008 GN=ECBD_4107 PE

Figure S8: Mass spectrometry analysis of gel band derived from SDS-PAGE. The protein was identified as outer membrane porin F (OmpF).

Accession	-10lgP	Coverage (%)	Coverage (%) Gel2 Band 1	Area Gel2 Band 1	#Peptides	#Unique	#Spec Gel2 Band 1	PTM	Avg. Mass	Description
A0A140NAP3	430.36	73	73	1.4645E9	90	90	455	Y	35541	L-arabinose-binding periplasmic protein OS=Escherichia coli (strain B / BL21-DE3) OX=469008 GN=ECBD_1740 PE=3 SV=1
A0A140N9C2	321.83	70	70	5.738E7	40	40	92	Y	37628	Sulfate ABC transporter, periplasmic sulfate-binding protein OS=Escherichia coli (strain B / BL21-DE3) OX=469008 GN=ECBD_1256 PE=3 SV=1
A0A140N775	308.39	82	82	6.0742E7	26	26	54	Y	32337	Malate dehydrogenase OS=Escherichia coli (strain B / BL21-DE3) OX=469008 GN=mdh PE=3 SV=1
A0A140NFH4	292.40	71	71	3.1557E7	18	18	38	Y	31944	Maltose operon periplasmic OS=Escherichia coli (strain B / BL21-DE3) OX=469008 GN=ECBD_3998 PE=4 SV=1
A0A140N9N8	284.70	61	61	2.3622E7	22	22	30	Y	40826	Putrescine-binding periplasmic protein OS=Escherichia coli (strain B / BL21-DE3) OX=469008 GN=ECBD_2740 PE=3 SV=1
A0A140NCN3	255.25	67	67	9.8802E6	16	16	22	Y	38867	Putrescine-binding periplasmic protein OS=Escherichia coli (strain B / BL21-DE3) OX=469008 GN=ECBD_2476 PE=3 SV=1
A0A140N9H6	243.15	54	54	8.8296E6	20	20	30	Y	33873	Uncharacterized protein OS=Escherichia coli (strain B / BL21-DE3) OX=469008 GN=ECBD_2042 PE=4 SV=1
A0A140NDV9	243.13	52	52	1.2203E7	14	14	20	Y	35249	Transaldolase OS=Escherichia coli (strain B / BL21-DE3) OX=469008 GN=tal PE=3 SV=1
A0A140NEX7	226.74	45	45	8.7194E6	12	12	23	Y	34313	Periplasmic binding protein/LacI transcriptional regulator OS=Escherichia coli (strain B / BL21-DE3) OX=469008 GN=ECBD_3806 PE=4 SV=1
A0A140N2V7	224.23	41	41	7.4144E6	12	12	17	Y	37047	Extracellular solute-binding protein family 3 OS=Escherichia coli (strain B / BL21-DE3) OX=469008 GN=ECBD_0476 PE=3 SV=1
A0A140NHP0	222.21	48	48	8.2959E6	13	13	18	Y	34080	Branched-chain-amino-acid aminotransferase OS=Escherichia coli (strain B / BL21-DE3) OX=469008 GN=ilvE PE=3 SV=1
A0A140N783	219.93	59	59	1.0132E7	17	17	25	Y	35533	Glyceraldehyde-3-phosphate dehydrogenase OS=Escherichia coli (strain B / BL21-DE3) OX=469008 GN=ECBD_1865 PE=3 SV=1
A0A140N8D8	206.79	31	31	1.6343E6	9	9	11	Y	39418	Histidinol-phosphate aminotransferase OS=Escherichia coli (strain B / BL21-DE3) OX=469008 GN=hisC PE=3 SV=1
A0A140ND51	205.10	40	40	1.2774E6	10	10	12	N	36163	Thiamine-binding periplasmic protein OS=Escherichia coli (strain B / BL21-DE3) OX=469008 GN=ECBD_3549 PE=3 SV=1
A0A140N5L9	201.58	52	52	1.5616E6	11	11	11	Y	34490	Cysteine synthase OS=Escherichia coli (strain B / BL21-DE3) OX=469008 GN=ECBD_1267 PE=3 SV=1
A0A140N4K7	201.48	45	45	6.7878E6	11	11	19	Y	35720	D-xylose ABC transporter, periplasmic substrate-binding protein OS=Escherichia coli (strain B / BL21-DE3) OX=469008 GN=ECBD_0168 PE=4
A0A140NAE3	192.10	32	32	5.4489E5	6	6	7	Y	35315	Glyoxylate/hydroxypyruvate reductase A OS=Escherichia coli (strain B / BL21-DE3) OX=469008 GN=ghrA PE=3 SV=1
A0A140N8E1	189.62	24	24	7.1943E5	5	5	5	Y	41118	Phosphoglycerate kinase OS=Escherichia coli (strain B / BL21-DE3) OX=469008 GN=pgk PE=3 SV=1
A0A140N801	180.97	41	41	4.9998E5	9	9	10	Y	35561	Glutathione synthetase OS=Escherichia coli (strain B / BL21-DE3) OX=469008 GN=gshB PE=3 SV=1
A0A140NCD0	179.35	32	32	1.1042E6	8	8	10	N	43388	Maltodextrin-binding protein OS=Escherichia coli (strain B / BL21-DE3) OX=469008 GN=ECBD_4002 PE=1 SV=1
A0A140NEY3	177.51	39	39	3.3701E5	7	7	8	Y	34643	Porphobilinogen deaminase OS=Escherichia coli (strain B / BL21-DE3) OX=469008 GN=hemC PE=3 SV=1
A0A140N6W0	176.96	32	32	2.5272E6	10	10	13	Y	43284	Elongation factor Tu OS=Escherichia coli (strain B / BL21-DE3) OX=469008 GN=tuf PE=1 SV=1
A0A140NCI6	176.96	32	32	2.5272E6	10	10	13	Y	43314	Elongation factor Tu OS=Escherichia coli (strain B / BL21-DE3) OX=469008 GN=tuf PE=3 SV=1
A0A140NDI4	176.19	41	41	4.755E5	11	11	11	Y	39840	Phosphoserine aminotransferase OS=Escherichia coli (strain B / BL21-DE3) OX=469008 GN=serC PE=3 SV=1
A0A140SS92	172.36	50	50	3.0695E6	12	12	14	Y	36659	Sulfate ABC transporter, periplasmic sulfate-binding protein OS=Escherichia coli (strain B / BL21-DE3) OX=469008 GN=ECBD_4107 PE=3 SV=1
A0A140NCS9	169.41	36	36	1.0175E6	9	9	13	Y	36649	Ribose-phosphate pyrophosphokinase OS=Escherichia coli (strain B / BL21-DE3) OX=469008 GN=prs PE=3 SV=1
A0A140N6M4	167.29	43	43	1.0807E6	11	11	13	Y	34893	ADP-L-glycero-D-manno-heptose-6-epimerase OS=Escherichia coli (strain B / BL21-DE3) OX=469008 GN=hldD PE=3 SV=1
A0A140NCB1	167.06	43	43	5.5398E5	9	9	12	N	35171	Alcohol dehydrogenase zinc-binding domain protein OS=Escherichia coli (strain B / BL21-DE3) OX=469008 GN=ECBD_3982 PE=3 SV=1

Figure S9: Mass spectrometry analysis of gel band derived from SDS-PAGE. The protein was identified as the L-Arabinose binding periplasmic protein (AraF).

Accession	-10lgP	Coverage (%)	Coverage (%) Gel2_Band1	Area Gel2_Band1	#Peptides	#Unique	#Spec Gel2_Band1	PTM	Avg. Mass	Description
A0A140N5U8	429.65	86	86	2.2519E9	87	82	249	Y	27992	Cationic amino acid ABC transporter, periplasmic binding protein OS=Escherichia coli (strain B / BL21-DE3) OX=469008 GN=ECBD_1349 PE=3 SV=1
A0A140N856	400.21	80	80	5.1952E8	70	64	115	Y	28483	Cationic amino acid ABC transporter, periplasmic binding protein OS=Escherichia coli (strain B / BL21-DE3) OX=469008 GN=ECBD_1350 PE=3 SV=1
A0A140NEL5	291.73	77	77	8.0121E7	33	33	52	Y	26972	Triosephosphate isomerase OS=Escherichia coli (strain B / BL21-DE3) OX=469008 GN=tpiA PE=3 SV=1
A0A140N9D9	289.88	64	64	1.9739E8	31	31	53	N	28556	2,3-bisphosphoglycerate-dependent phosphoglycerate mutase OS=Escherichia coli (strain B / BL21-DE3) OX=469008 GN=gpmA PE=3 SV=1
A0A140N9F1	286.78	52	52	2.3913E7	26	26	36	Y	48542	Long-chain fatty acid transport protein OS=Escherichia coli (strain B / BL21-DE3) OX=469008 GN=ECBD_1316 PE=3 SV=1
A0A140NAP3	286.71	61	61	3.352E7	28	28	42	Y	35541	L-arabinose-binding periplasmic protein OS=Escherichia coli (strain B / BL21-DE3) OX=469008 GN=ECBD_1740 PE=3 SV=1
A0A140NFM7	285.20	59	59	9.2137E7	27	27	44	Y	24730	50S ribosomal protein L1 OS=Escherichia coli (strain B / BL21-DE3) OX=469008 GN=rplA PE=3 SV=1
A0A140N4K1	284.13	65	65	7.2369E7	31	31	48	Y	25983	30S ribosomal protein S3 OS=Escherichia coli (strain B / BL21-DE3) OX=469008 GN=rpsC PE=3 SV=1
A0A140NFD2	277.79	68	68	2.5968E7	23	22	30	N	29418	Lipoprotein OS=Escherichia coli (strain B / BL21-DE3) OX=469008 GN=ECBD_3421 PE=3 SV=1
A0A140N9V3	276.58	55	55	2.5793E7	20	20	30	Y	27190	Cationic amino acid ABC transporter, periplasmic binding protein OS=Escherichia coli (strain B / BL21-DE3) OX=469008 GN=ECBD_2812 PE=3 SV=1
A0A140NA83	264.34	64	64	4.0261E7	24	24	44	Y	27864	Enoyl-[acyl-carrier-protein] reductase [NADH] OS=Escherichia coli (strain B / BL21-DE3) OX=469008 GN=ECBD_2329 PE=1 SV=1
A0A140N8Q2	262.69	66	66	1.5512E7	18	18	28	Y	27477	3-dehydroquinate dehydratase OS=Escherichia coli (strain B / BL21-DE3) OX=469008 GN=aroD PE=3 SV=1
A0A140NCI3	256.26	59	59	5.4265E7	19	19	32	Y	25493	Phage shock protein A, PspA OS=Escherichia coli (strain B / BL21-DE3) OX=469008 GN=ECBD_2313 PE=3 SV=1
A0A140N9M6	251.96	58	58	5.1879E6	18	18	29	Y	33375	Extracellular solute-binding protein family 3 OS=Escherichia coli (strain B / BL21-DE3) OX=469008 GN=ECBD_2996 PE=4 SV=1
A0A140SSB0	244.94	51	51	2.4465E7	16	16	24	Y	27287	Uridine phosphorylase OS=Escherichia coli (strain B / BL21-DE3) OX=469008 GN=ECBD_4198 PE=3 SV=1
A0A140NCM6	238.69	59	59	1.3787E7	13	13	23	Y	27204	3'(2'),5'-bisphosphate nucleotidase CysQ OS=Escherichia coli (strain B / BL21-DE3) OX=469008 GN=cysQ PE=3 SV=1
A0A140N9V2	236.38	56	56	3.844E6	16	16	17	Y	28724	Tryptophan synthase alpha chain OS=Escherichia coli (strain B / BL21-DE3) OX=469008 GN=trpA PE=3 SV=1
A0A140NA86	236.10	74	74	3.1978E7	13	13	24	Y	22516	Penicillin-binding protein activator LpoB OS=Escherichia coli (strain B / BL21-DE3) OX=469008 GN=lpoB PE=3 SV=1
A0A140NCF6	234.45	42	42	4.2198E6	13	13	14	Y	28262	Cell division coordinator CpoB OS=Escherichia coli (strain B / BL21-DE3) OX=469008 GN=cpoB PE=3 SV=1
A0A140NGH9	232.00	53	53	3.2709E6	13	13	20	Y	27748	Deoxyribose-phosphate aldolase OS=Escherichia coli (strain B / BL21-DE3) OX=469008 GN=deoC PE=3 SV=1
A0A140NFK2	227.56	69	69	4.9855E7	17	17	30	Y	26744	30S ribosomal protein S2 OS=Escherichia coli (strain B / BL21-DE3) OX=469008 GN=rpsB PE=3 SV=1
A0A140NBF8	225.85	58	58	4.5242E6	14	13	22	Y	26857	Cationic amino acid ABC transporter, periplasmic binding protein OS=Escherichia coli (strain B / BL21-DE3) OX=469008 GN=ECBD_2734 PE=3 SV=1
A0A140N9H6	225.24	64	64	4.4914E6	20	20	21	Y	33873	Uncharacterized protein OS=Escherichia coli (strain B / BL21-DE3) OX=469008 GN=ECBD_2042 PE=4 SV=1
A0A140NDW9	218.23	46	46	1.1451E7	17	17	25	Y	29892	2,3,4,5-tetrahydropyridine-2,6-dicarboxylate N-succinyltransferase OS=Escherichia coli (strain B / BL21-DE3) OX=469008 GN=dapD PE=3 SV=1

Figure S10: Mass spectrometry analysis of gel band derived from SDS-PAGE. The protein was identified as a cationic amino acid ABC transporter protein. Two different bands were identified with the same sequence, indicating protein homologues.

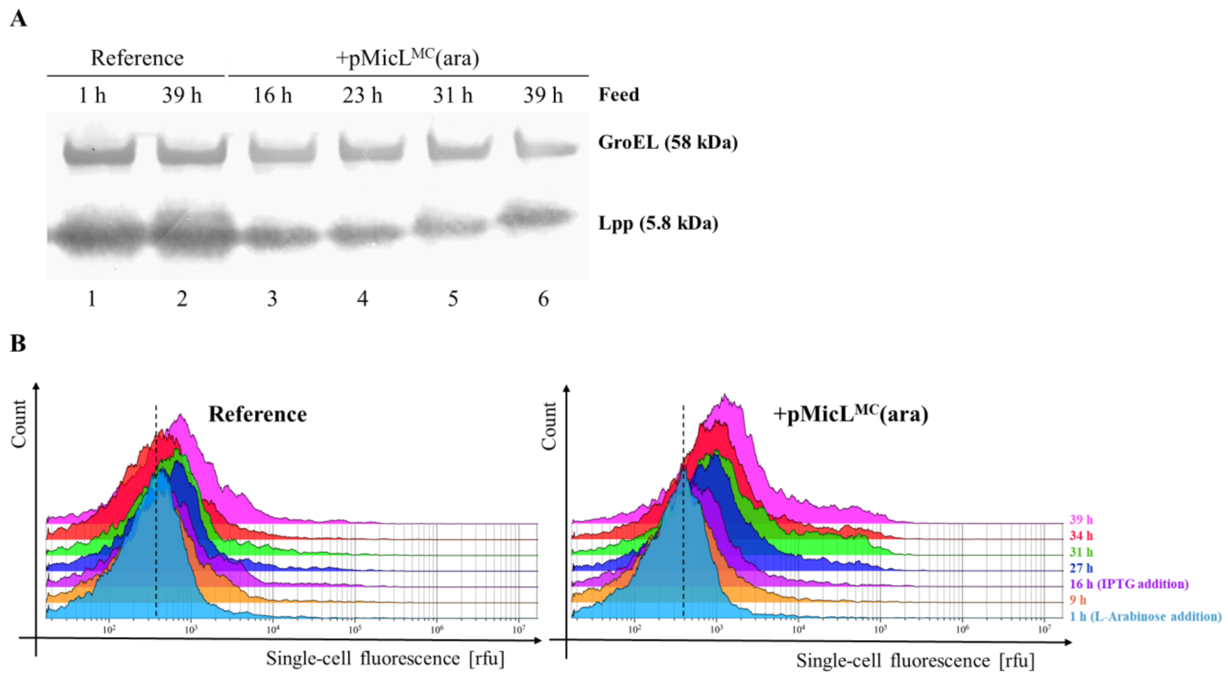

Figure S11: Western blot analysis (**A**) and flow cytometry analysis (**B**) of BΔ*ara*<oCASPON-SST> (Reference) and BΔ*ara*<oCASPON-SST> pMicL^{MC}(ara) during stirred-tank bioreactor cultivations with linear feed profile. L-Arabinose and IPTG were added to the cultivation after 1 and 16 hours, respectively. GroEL was stained as loading control. Single-cell fluorescence was analyzed by incubating cell suspension with SYTOX™ green and subsequent flow cytometry analysis.
